# The adaptor protein TRAF3 is an immune checkpoint that inhibits myeloid-derived suppressor cell expansion

**DOI:** 10.3389/fimmu.2023.1167924

**Published:** 2023-05-03

**Authors:** Sining Zhu, Almin I. Lalani, Juan Jin, Derek Sant’Angelo, Lori R. Covey, Kebin Liu, Howard A. Young, Suzanne Ostrand-Rosenberg, Ping Xie

**Affiliations:** ^1^ Department of Cell Biology and Neuroscience, Rutgers University, Piscataway, NJ, United States; ^2^ Graduate Program in Cellular and Molecular Pharmacology, Rutgers University, Piscataway, NJ, United States; ^3^ Department of Pharmacology, Anhui Medical University, Hefei, Anhui, China; ^4^ Child Health Institute of New Jersey, Rutgers University, New Brunswick, NJ, United States; ^5^ Department of Pediatrics, Rutgers Robert Wood Johnson Medical School, Rutgers University, New Brunswick, NJ, United States; ^6^ Rutgers Cancer Institute of New Jersey, New Brunswick, NJ, United States; ^7^ Department of Biochemistry and Molecular Biology, Medical College of Georgia, Augusta, GA, United States; ^8^ Laboratory of Cancer Immunometabolism, Center for Cancer Research, National Cancer Institute at Frederick, National Institutes of Health, Frederick, MD, United States; ^9^ Department of Biological Sciences, The University of Maryland, Baltimore County, Baltimore, MD, United States

**Keywords:** myeloid-derived suppressor cells, chronic inflammation, TRAF3, CCL22, STAT3

## Abstract

Myeloid-derived suppressor cells (MDSCs) are aberrantly expanded in cancer patients and under other pathological conditions. These cells orchestrate the immunosuppressive and inflammatory network to facilitate cancer metastasis and mediate patient resistance to therapies, and thus are recognized as a prime therapeutic target of human cancers. Here we report the identification of the adaptor protein TRAF3 as a novel immune checkpoint that critically restrains MDSC expansion. We found that myeloid cell-specific *Traf3*-deficient (M-*Traf3* ^-/-^) mice exhibited MDSC hyperexpansion during chronic inflammation. Interestingly, MDSC hyperexpansion in M-*Traf3* ^-/-^ mice led to accelerated growth and metastasis of transplanted tumors associated with an altered phenotype of T cells and NK cells. Using mixed bone marrow chimeras, we demonstrated that TRAF3 inhibited MDSC expansion *via* both cell-intrinsic and cell-extrinsic mechanisms. Furthermore, we elucidated a GM-CSF-STAT3-TRAF3-PTP1B signaling axis in MDSCs and a novel TLR4-TRAF3-CCL22-CCR4-G-CSF axis acting in inflammatory macrophages and monocytes that coordinately control MDSC expansion during chronic inflammation. Taken together, our findings provide novel insights into the complex regulatory mechanisms of MDSC expansion and open up unique perspectives for the design of new therapeutic strategies that aim to target MDSCs in cancer patients.

## Highlights

- The adaptor protein TRAF3 is an immune checkpoint of MDSC expansion.
- Young adult M-*Traf3* ^-/-^ mice with chronic inflammation exhibit accelerated growth and metastasis of transplanted lymphomas.
- A novel TRAF3-CCL22-CCR4-G-CSF axis acts in inflammatory macrophages and monocytes to restrain MDSC expansion during chronic inflammation.
- TRAF3 specifically inhibits the GM-CSF-STAT3 signaling axis in MDSCs.

## Introduction

Cancer immunotherapies, including immune checkpoint inhibitors, adoptive transfer of *ex vivo* expanded tumor-specific T cells or chimeric antigen receptor T cells (CAR-T), and cancer vaccines, are recognized as breakthroughs in cancer treatments (1). In particular, clinical success of the immune checkpoint PD-L1/PD1 inhibitors has been demonstrated in many different types of human cancers (1). However, current cancer immunotherapies are only effective in a subset of patients and immunosuppression has been identified as one major obstacle of cancer immunotherapies (2–4). Myeloid-derived suppressor cells (MDSCs), a heterogeneous population of immature myeloid cells, are expanded in most human cancers and act as a core orchestrator of the immunosuppressive network in cancer patients (2–4). MDSCs are crucial drivers of chronic inflammation and potent suppressors of immune responses, contributing to patient resistance to immunotherapy, chemotherapy, and radiotherapy (4–8). Furthermore, MDSCs facilitate cancer metastasis by establishing premetastatic niches and by inducing cancer stem cell formation, mutagenesis, angiogenesis, and invasion (8, 9). MDSC-targeting drugs and approaches are currently under active investigation in clinical trials in combination with various immune checkpoint inhibitors, chemotherapy, and radiotherapy (4–6). Thus, there is a pressing need to better understand the mechanisms controlling MDSC expansion in order to develop more precise MDSC-targeting strategies to improve patient outcome. Here we report the identification of a novel regulator of MDSC expansion: a cytoplasmic adaptor protein termed tumor necrosis factor receptor (TNFR)-associated factor 3 (TRAF3) (10).

Being a member of the TRAF family of adaptor proteins and E3 ubiquitin ligases, TRAF3 is utilized by a variety of immune receptors for signaling, including the TNF-R superfamily, pattern recognition receptors (PRRs), and cytokine receptors, among others (11–13). TRAF3 has been recognized as a pivotal regulator of the adaptive immune system, including B cell survival and homeostasis (14–19), antibody class switching and B cell anergy (20, 21), T cell activation and memory responses (22, 23), and regulatory T cell (Treg) development and effector functions (24, 25). In particular, increasing evidence indicates that TRAF3 is a tumor suppressor in B lymphocytes and that its absence in B cells is frequently associated with the development of B cell neoplasms, including non-Hodgkin lymphoma and multiple myeloma (11, 13, 26). Beyond the adaptive immune system, we recently investigated the function of TRAF3 in the innate immune system by studying a mouse model that has the *Traf3* gene specifically deleted in myeloid cells (M-*Traf3* ^-/-^ mice). We reported that specific ablation of TRAF3 in myeloid cells does not affect the maturation or homeostasis of macrophages and neutrophils in young adult naïve mice (27). Interestingly, aging M-*Traf3* ^-/-^ mice exhibit spontaneous chronic inflammation and tumor development, which are consistently associated with a striking expansion of CD11b+Gr1+ myeloid cells (27, 28). Thus, TRAF3 plays diverse and indispensable roles in both adaptive and innate immunity through distinct context-dependent mechanisms.

In the present study, we identify TRAF3 as a critical immune checkpoint in myeloid cells that restrains MDSC expansion during chronic inflammation. Our findings provide novel insights into the complex cellular and molecular mechanisms underpinning the regulation of MDSC expansion. This knowledge will open up unique avenues for the design of new strategies that aim to target or exploit MDSCs in different clinical settings.

## Methods

### Mice and cell lines

*Traf3* ^flox/flox^ and *Traf3* ^flox/flox^LysM^+/Cre^ (M-*Traf3* ^-/-^) mice on the C57BL/6J genetic background were generated as previously described (27). Mouse ear tissues were screened by genomic PCR using primer sets (FC3 + BT6) (14), (Lys-Com + Lys-WT), and (Lys-Com + Lys-Cre) (27). Deletion of exons 1 and 2 of the *Traf3* gene in MDSCs and macrophages was detected by genomic PCR using primers U7 and BT6 as previously described (14). All experimental mice for this study were produced by breeding *Traf3* ^flox/flox^ mice with *Traf3* ^flox/flox^LysM^+/Cre^ mice. *Traf3* ^flox/flox^ littermates (LMC) were used as controls for all experiments. C57.SJL (CD45.1) mice were purchased from Jackson Laboratory (Stock No: 002014, Bar Harbor, Maine). *Traf3* ^flox/flox^ mice (CD45.2) were bred with C57.SJL mice to generate wild type (WT) donor mice (CD45.1/CD45.2) for mixed bone marrow (BM) chimera experiments. All mice were kept in specific pathogen-free conditions in the Animal Facility at Rutgers University and were used in accordance with NIH guidelines under an animal protocol (PROTO999900371) approved by the Animal Care and Use Committee of Rutgers University. Mice were monitored daily for signs of illness or discomfort, including weight loss, enlarged lymph nodes or abdomen, labored breathing, hunched posture and paralysis. Equal numbers of male and female mice were used in this study.

Mouse B-cell lymphoma cell lines 291-6.1.1-8 (291-6) and 291-7.1.2 Asc C1 (291-7) were generated and cultured as described previously (16, 29). Briefly, primary B-cell lymphomas were harvested from the spleen of an M-*Traf3* ^-/-^ mouse (mouse ID: 291-6) (27) and from the ascites of another M-*Traf3* ^-/-^ mouse (mouse ID: 291-7) (27), and then passaged in NOD SCID mice once. Lymphoma cells harvested from the transplanted NOD SCID mice were subsequently plated in 24-well plates at 5x10^5^ cells/well in mouse B cell media (14) containing 10% FCS. After being cultured for 1 month, several actively proliferating clones were expanded, passaged, and frozen. The 291-6.1.1-8 and 291-7.1.2 Asc C1 clones had been cultured for 5 months without obvious changes in morphology or growth rate and were used for lymphoma transplantation experiments. These two cell lines were characterized as B220+IgG+ by flow cytometry. Both cell lines exhibit the typical morphology of DLBCL and contain somatic hypermutations (SHM) in the IgH VDJ region. The human embryonic kidney 293T cell line was purchased from American Type Culture Collection (ATCC, Manassas, VA) and was cultured according to the manufacturer’s protocol.

### Antibodies and reagents

Fluorochrome-labeled antibodies (Abs) against mouse CD11b, Gr1, Ly6C, Ly6G, CD45R (B220), CD3, CD45.1, CD45.2, F4/80, CD11c, MHC class II, CD4, CD8, CD115, CD169, NK1.1, CD49b, CD62L, CD69, CX3CR1, CCR2, CCR4, PD-L1, CD38, KLRG1, NKp46, and Sca1 were purchased from BioLegend (San Diego, CA) and eBioscience (San Diego, CA). Anti-BrdU, anti-p-STAT3 (pY705), anti-p-STAT5 (pY694), anti-p-ERK1/2 (pT202/pY204), heat-killed Bacillus Calmette-Guérin (BCG, *M. tuberculosis* H37 Ra), and Matrigel Matrix were from BD Biosciences (San Jose, CA). Mouse Cytokine Array Kit Panel A, ELISA Ab pairs for mouse G-CSF, CXCL-13, S100A8/A9, and CCL22, as well as neutralizing Abs for mouse G-CSF and CXCL-13 were obtained from R&D (Minneapolis, MN). Anti-mouse HMGB1 ELISA kit was purchased from Biomatik (Wilmington, DE). Rabbit Abs for HA tag and V5 tag were from Cell Signaling Technology (Beverly, MA). Mouse monoclonal Abs to SBP tag were purchased from EMD Millipore Corp (Burlington, MA). HRP-labeled secondary Abs were from Jackson ImmunoResearch Laboratories, Inc. (West Grove, PA).

Incomplete Freund’s Adjuvant, heparin, 5′-bromo-2′-deoxyuridine (BrdU), LPS, Stattic, Baricitinib, anti-FLAG M2 Affinity Gel, rabbit Abs for FLAG tag, FLAG peptides, and Microcon-10kDa spin columns were purchased from Sigma (St. Louis, MO). Mouse MDSC purification kits and anti-phycoerythrin (PE) magnetic beads were from Miltenyi Biotec Inc. (Auburn, CA). Tissue culture supplements including stock solutions of sodium pyruvate, L-glutamine, non-essential amino acids, and HEPES (pH 7.55) were from Invitrogen (Carlsbad, CA). Lymphocyte Separation Medium was purchased from Lonza (Morristown, NJ). GM-CSF, G-CSF, M-CSF, IL-4, and IL-6 were from PeproTech (Rocky Hill, NJ). Hyaluronidase, Collagenase type 3, and type 4 were from Worthington Biochemical Corp (Lakewood, NJ). DNase I, Phosphatase Inhibitor Mini Tablets, and Streptavidin-Sepharose beads were purchased from Pierce (Rockford, IL). EDTA-free Protease Inhibitor Cocktail Tablets were obtained from Roche Diagnostics Corp (Indianapolis, IN). SureBlue™ TMB Microwell Peroxidase Substrate Kit was purchased from KPL Inc. (Milford, MA). DNA plasmids of STAT3, PTP1B, and JAK2 were purchased from Addgene (Watertown, MA). DNA oligonucleotide primers were obtained from Integrated DNA Technologies (Coralville, IA).

### Flow cytometry

Single cell suspensions were made from the spleen, draining lymph node (DLN), BM, peripheral blood cells (PBL), and tumor. Immunofluorescence staining and FACS analyses were performed as previously described (14, 16, 21). Erythrocytes from the spleen were depleted with ACK lysis buffer. PBL (~100 µl) was collected by retro-orbital bleeding with a calibrated micropipette coated with heparin into a tube containing 15 µl of heparin (10 KU/ml) to prevent coagulation. Red blood cells (RBC) were depleted from PBL with RBC Lysis Buffer (BioLegend), or alternatively, peripheral blood mononuclear cells (PBMCs) were prepared from PBL using Lymphocyte Separation Medium (Lonza) following the manufacturer**’**s protocols. Cells (1x10^6^) were blocked with rat serum and FcR blocking Ab (2.4G2), and then incubated with various Abs conjugated to different fluorochromes for multiple color fluorescence surface staining. Intracellular markers were stained after fixation and permeabilization of cells using an Intracellular or Phospho-Flow Staining kit (BD Biosciences). BrdU labeling on cellular DNA was measured using the BrdU Flow Kit (BD Biosciences). FACS data were acquired on a Northern Lights spectral flow cytometer (Cytek, Fremont, CA) or a FACSCalibur (Becton Dickinson, Mountain View, CA). The results were analyzed using the FlowJo software (TreeStar, San Carlos, CA) (21).

### Purification of splenic MDSCs using magnetic sorting

MDSCs were purified from splenocytes by first depleting B cells and T cells using anti-B220 and anti-CD90.2 beads (Miltenyi Biotec), and then collecting Gr1+ MDSCs from the B220-CD90.2- fraction using anti-Gr1-PE followed by anti-PE beads (Miltenyi Biotec). The purity of prepared MDSC populations was verified to be greater than 95% of B220-CD3-CD11b+Gr1+ as determined by FACS.

### MDSC morphological examination

Morphological characterization of MDSC subtypes was performed on cytospin preparations of splenic MDSCs purified from aging M-*Traf3* ^-/-^ mice with chronic inflammation or B-cell lymphomas. Wright-Giemsa staining of cytospin slides was carried out using a Diff-Quick staining kit (VWR, Radnor, PA) following the manufacturer’s procedures. Bright field micrographs of stained slides were taken using a microscope (Olympus BX-51, Olympus America Inc., Center Valley, PA).

### Co-culture of purified MDSCs and syngeneic CD8 T cells

CD8 T cells were purified from naïve LMC mice using a CD8+ T Cell Purification Kit (Miltenyi Biotec) following the manufacturer’s instructions. Purified CD8 T cells were labeled with 0.5 µM of a cell proliferation dye eFluor 670 (eBioscience) at 37°C for 10 min according to the manufacturer’s protocol (30). Labeled cells were cultured in 24-well plates pre-coated with 10 µg/ml of anti-CD3 antibody (BioLegend), in the absence or presence of 2 µg/ml of anti-CD28 antibody (BioLegend) or purified MDSCs (at a 1:1 ratio to T cells), at 37°C for 4 days. The proliferation of gated CD8+ T cells was monitored by flow cytometric analysis of the eFluor 670 dye dilution.

### Heat-killed BCG-induced chronic inflammation in mice

Chronic inflammation was induced in young adult (2-4-month-old) mice by repeated injections of heat-killed BCG as previously described (31). Briefly, mice were *s.c.* injected with heat-killed BCG (100 μg/100 μl/mouse) at 1-week intervals. The first two injections were administrated as a mixture of BCG/PBS and incomplete Freud adjuvant (IFA) at a 1:1 ratio emulsified by vortexing for 15 minutes. The third BCG injection was in PBS only. Unless stated otherwise, cells were collected on day 2 after each injection.

### Tumor transplantation

Young adult (2-4-month-old) LMC and M-*Traf3* ^-/-^ mice were injected *s*.*c*. with 5x10^6^ cells of 291-6 and 291-7 B lymphoma cell lines (mixed at a 1:1 ratio) for tumor transplantation. For mice that received 3 injections of heat-killed BCG, tumor transplantation was performed on day 2 after the third BCG injection. For end-point analysis, DLBCL cells were injected in 100 µl PBS. For analysis at day 7 post transplantation, DLBCL cells were injected in 300 µl of 50% Matrigel in PBS. Transplanted mice were monitored daily for tumor growth and signs of failing health (32). At the end-point of tumor growth or day 7 post transplantation, the spleen, DLN, and tumors were harvested from each mouse for FACS analysis.

### Tumor dissociation for FACS analysis

Dissected lymphomas or subcutaneous Matrigel plugs were cut into small (< 3 mm) pieces and incubated in 1-5 ml of enzyme dissociation solution (PBS containing 2.5 mg/ml of Hyaluronidase, 2.5 mg/ml of Collagenase Type 3, 2.5 mg/ml of Collagenase Type 4, and 100 μg/ml of DNase I) at 37°C with shaking for 40 min, vortexing every 5 minutes during the incubation. Dissociated tumor cells were filtered through a 70 μm cell strainer to remove residual tissues and then washed twice with PBS before immunofluorescence staining for FACS analysis.

### Mixed BM chimeras

C57.SJL (CD45.1) recipient mice (8-12-wk-old) were subjected to whole-body X-ray radiation with a single dose of 1,100 rads at 24 h before BM transfer. The radiation was delivered at the rate of 0.28 Gy/min using an aluminum filter at 100 kVp (Faxitron Cabinet X-ray System, Faxitron X-ray Corp., Wheeling, IL) (33). Total BM cells were harvested from the femurs and tibias of donor WT (CD45.1/CD45.2) and M-*Traf3* ^-/-^ (CD45.2) mice (8-12-wk-old) and mixed at a 1:1 ratio. Each C57.SJL recipient mouse was injected *i.v.* with 1×10^7^ of mixed BM cells in 100 µl PBS. At 8 weeks after reconstitution, the chimerism in PBL was examined by FACS. A cohort of mixed BM chimeras were subjected to repeated injections of heat-killed BCG and analyzed on day 2 after the 3^rd^ BCG injection. Another cohort of naïve BM chimeras were directly sacrificed and analyzed at the same time with the BCG-treated chimeras. The ratios of M-*Traf3* ^-/-^ (CD45.2) *versus* WT (CD45.1/CD45.2) CD11b+Gr1+ cells, B cells, and T cells in the spleen, PBL, and BM were analyzed by FACS and compared between naïve and BCG-treated BM chimeras.

### Mouse cytokine protein array assay and ELISA

Sera were collected from LMC and M-*Traf3* ^-/-^ mice at day 1 or day 2 after the 2^nd^ and 3^rd^ BCG injections. Serum levels of 40 cytokines and chemokines were measured using a Mouse Cytokine Array Panel A kit (R&D, Minneapolis, MN) following the manufacturer’s instructions (27). Images of the blots were acquired and quantitative analyses of cytokine/chemokine spots were performed using a low-light imaging system (LAS-4000 mini, FUJIFILM Medical Systems USA, Inc., Stamford, CT) (27). Concentrations of G-CSF, CXCL-13, S100A8/A9, HMGB1, and CCL22 in mouse sera were determined by quantitative ELISA using specific coating Abs and biotinylated detection Abs (R&D or Biomatik) following the manufacturer’s protocols.

### *In vivo* BrdU labeling

Gender-matched, young adult (8-12-wk-old) mice were *s.c.* injected with heat-killed BCG (a mixture of BCG/PBS and IFA at a 1:1 ratio; for each injection, 100 μg/100 µl/mouse) twice at a 1-week interval. Mice were *i.p.* injected with BrdU (3 mg in 0.3 ml of PBS) twice at 19 h and 16 h before sacrifice. Mice were euthanized on day 2 after the 2^nd^ BCG injection. BrdU+ cells were detected by intracellular staining with an APC-conjugated anti-BrdU antibody using the BrdU Flow Kit (BD Pharmingen™) following the manufacturer’s protocol.

### *In vivo* neutralization of G-CSF and CXCL-13

LMC and M-*Traf3* ^-/-^ mice (8-12-wk-old) were injected *i.p.* with anti-G-CSF neutralizing antibody (25 μg in 100 μl of PBS/mouse, R&D), anti-CXCL-13 neutralizing Ab (10 μg in 100 μl of PBS/mouse, R&D), or Ig isotype control, beginning at 1 day after the 1^st^ injection of heat-killed BCG and continuing thrice weekly for 2 weeks. On day 2 after the 3^rd^ BCG injection, spleens and BM cells were harvested from the treated mice for FACS analysis.

### G-CSF production and chemokine receptor expression in cultured BM cells

BM cells were harvested from the femurs of naïve LMC and M-*Traf3* ^-/-^ mice (2-4-month-old) and cultured in the BM cell medium (RPMI 1640 medium containing 10% FBS, 1 mM sodium pyruvate, 0.1 mM nonessential amino acids, 2 mM L-glutamine, 100 U/ml penicillin, 100 µg/ml streptomycin, and 10 mM HEPES, pH 7.55), in the absence or presence of 10 ng/ml GM-CSF and 100 ng/ml LPS for 24 h. Production of G-CSF was detected by intracellular staining with Alexa Fluor 647-anti-G-CSF and expression of chemokine receptors (CCR2, CX3CR1, and CCR4) were determined by immunofluorescence surface staining, followed by FACS analysis.

### Real-time PCR analysis of BM-derived macrophages

BM cells were harvested from the femurs and tibias of LMC and M-*Traf3* ^-/-^ mice (8-12-wk-old). BMDMs were prepared as previously described (27) and cultured in the absence or presence of 100 ng/ml LPS for 2 or 24 h. Total cellular RNA was extracted using TRIzol reagent (Invitrogen, Carlsbad, CA) according to the manufacturer’s protocol. cDNA was prepared from RNA using the High Capacity cDNA Reverse Transcription Kit (Applied Biosystems, Foster City, CA). Quantitative real-time PCR of cDNA samples was performed using TaqMan primers and probe (FAM-labeled) specific for mouse *Ccl2*, *Ccl3*, *Ccl7*, *Ccl22*, or *Cx3cl1* (Applied Biosystems). Each reaction also included the primers and probe (VIC-labeled) specific for mouse *Actb*, which served as an endogenous control. Reactions were performed on a QuantStudio™ 3 Real-Time PCR System (96-well, 0.1ml Block, Applied Biosystems) (21). Real-time PCR data were analyzed using the QuantStudio™ Design and Analysis Software v1.5.2 (Applied Biosystems) (21). Relative mRNA expression levels of each gene were calculated using the comparative Ct method (ΔΔCt) as described (27).

### BM cell proliferation assay

BM cells were harvested from the femurs of naïve LMC and M-*Traf3* ^-/-^ mice (2-4-month-old) and labeled with 0.5 µM of a cell proliferation dye eFluor 670 (eBioscience) at 37°C for 10 min according to the manufacturer’s protocol (30). Cells were subsequently cultured in non-treated 24-well plates at 37°C for 3 days, in the absence or presence of 20 ng/ml GM-CSF alone or 10 ng/ml GM-CSF in combination with 10 ng/ml of IL-4, IL-6, G-CSF, or M-CSF. For the study of the effects of JAK/STAT inhibitors, cells were cultured with 20 ng/ml GM-CSF, alone or in combination with 1 µM of Stattic or Baricitinib, at 37°C for 3 days. The proliferation of gated CD11b+Gr1+ BM cells was determined by FACS analysis of the labeled eFluor 670 dye dilution.

### Analysis of GM-CSF early signaling events

Before stimulation with GM-CSF, BM cells or purified splenic MDSCs were cultured and rested in 5% FCS medium for 2 hours. BM cells were stimulated with 20 ng/ml GM-CSF at 37°C for 10 min followed by Phospho-Flow FACS analysis. Purified splenic MDSCs were stimulated with 10 ng/ml GM-CSF at 37°C and total cellular protein lysates were prepared at different time points (0-60 min) as previously described (29) for measurements of early signaling events by Western blot analysis. Western blot analysis was performed as previously described (29, 30). Briefly, proteins were separated by SDS-PAGE and immunoblotted with antibodies specific for phosphorylated (P-) or total STAT3 (Cat# 9131 and 4904 from Cell Signaling Technology [CST]), STAT5 (Cat# 9359 and 9363 from CST), ERK1/2 (Cat# 9101 and 9102 from CST), IκBα (Cat# 2859 and 9242 from CST), p50 NF-κB1 (Cat# sc-114 from Santa Cruz Biotechnology [SCB]), RelA (Cat# 3033 and 8242 from CST), c-Rel (Cat# sc-71 from SCB), p100/p52 NF-κB2 (Cat# 4882 from CST), RelB (Cat# sc-226 from SCB), Akt (Cat# 9271 and 9272 from CST), GSK3α/β (Cat# 9331 from CST), CREB (Cat# 9191 from CST), TRAF3 (Cat# sc-1828 from SCB), or actin (Cat# MAB1501R from Millipore). Images of immunoblots were acquired using a low-light imaging system (LAS-4000 mini, FUJIFILM Medical Systems USA, Inc., Stamford, CT) (30, 34).

### GM-CSF-induced TRAF3 degradation in CD11b+Gr1+ BM cells

BM cells were harvested from the femurs and tibias of LMC and M-Traf3^-/-^ mice (8-12-wk-old). B cells, T cells, and RBCs were depleted from BM cells using anti-B220, anti-CD19, anti-CD90.2, and anti-Ter119 magnetic beads (Miltenyi Biotec), and then Gr1+ BM cells were purified from the B220-CD19-CD90.2-Ter119- fraction using anti-Gr1-PE followed by anti-PE beads (Miltenyi Biotec). The purity of the resultant Gr1+ populations was verified to be greater than 92% of B220-CD19-CD3-Ter119-CD11b+Gr1+ as determined by FACS. Purified CD11b+Gr1+ cells were stimulated with 20 ng/ml GM-CSF at 37**°**C and total cellular protein lysates were prepared at different time points (0, 6 and 12 **h**) as previously described (29) for measurements of TRAF3 degradation by Western blot analysis.

### Co-immunoprecipitation

For co-immunoprecipitation studies, 293T cells were transfected with pUB-TRAF3-SBP-6xHis (18) or pRc/CMV-STAT3-FLAG (Addgene) alone or in combination with pJ3H-PTP1B-HA (Addgene), pcw107-JAK2-V5 (Addgene), or an empty expression vector pUB-Thy1.1 (18). At day 2 post transfection, cells (2 x 10^7^ cells/condition) were harvested and total cellular proteins were lysed in 1% CHAPS Lysis Buffer (1% CHAPS, 20 mM Tris, pH 7.4, 150 mM NaCl, 50 mM β-glycerophosphate, and 5% glycerol with freshly added 1 mM DTT, 1x EDTA-free Mini-complete protease inhibitor cocktail, and 1x Phosphatase Inhibitors) (34), sonicated, and then cleared by centrifugation at 10,000 g for 20 minutes at 4°C. The cleared lysates were immunoprecipitated with Streptavidin (SA)-Sepharose beads (Pierce) for TRAF3-SBP or anti-FLAG M2 Affinity Gel (Sigma) for STAT3-FLAG (18). Immunoprecipitates were washed 5 times with the Wash Buffer (34). For SA IP, immunoprecipitated proteins were directly resuspended in 2X SDS sample buffer (34). For FLAG IP, immunoprecipitated proteins were eluted with 50 μl of 100 μg/ml FLAG peptides (Sigma) five times. The FLAG eluates of each sample were combined and concentrated using a 10 kDa cut-off spin column (Sigma). The concentrated eluates were subsequently resuspended in 2X SDS sample buffer (34). All the immunoprecipitated proteins or concentrated eluates were boiled for 10 minutes, and then separated on SDS-PAGE for Western blot analyses.

### Statistics

Statistical analyses were performed using the Prism software (GraphPad, La Jolla, CA). Survival curves were generated using the Kaplan-Meier method and were compared using a log-rank (Mantel-Cox) test to determine whether differences are significant. For direct comparison of cell subset frequencies or numbers, cytokine or chemokine levels, or expression levels of specific proteins between LMC and M-*Traf3* ^-/-^ mice, statistical significance was determined with the unpaired *t* test for two-tailed data. For comparison of three or more groups of data, a one-way analysis of variance (ANOVA) was used to determine the statistical significance. *P* values less than 0.05 are considered significant (*), *P* values less than 0.01 are considered very significant (**), and *P* values less than 0.001 are considered highly significant (***).

## Results

### TRAF3 is a novel regulator of MDSC expansion

**Figure 1:**
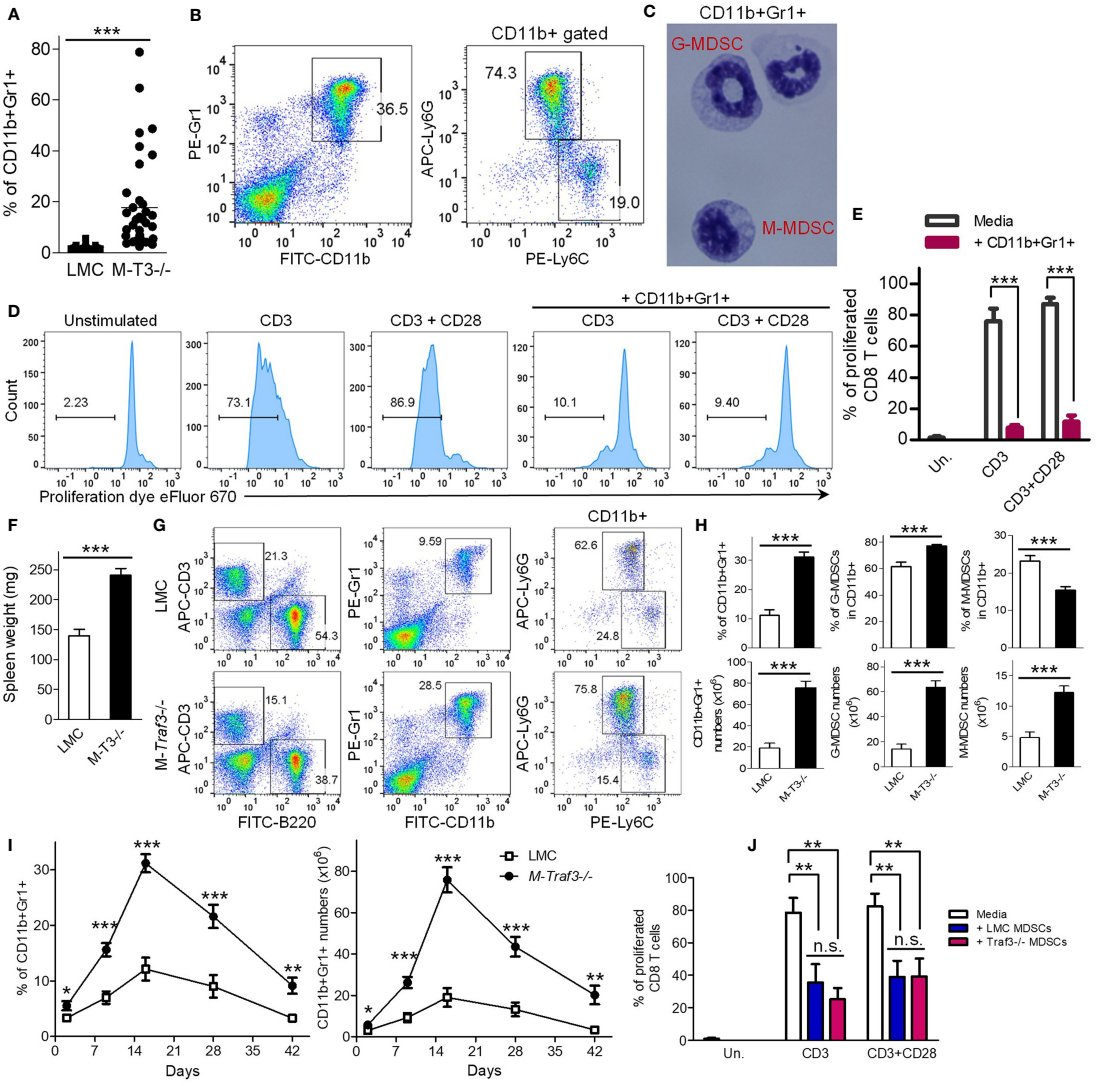
Aberrant expansion of splenic MDSCs in M-*Traf3* ^-/-^ mice. **(A–E)** Striking expansion of MDSCs in aging M-*Traf3* ^-/-^ mice (15-22-month-old) with spontaneous chronic inflammation or tumors. **(A)** Increased percentage of splenic CD11b+Gr1+ cells in aging M-*Traf3* ^-/-^ mice with diseases (n=36/group). **(B)** Representative FACS profiles of splenocytes stained for CD11b, Gr1, Ly6C, or Ly6G. **(C)** Morphology of purified CD11b+Gr1+ cells determined by Wright-Giemsa staining of cytospin slides. **(D)** Potent suppressive activity of splenic CD11b+Gr1+ cells purified from aging M-*Traf3* ^-/-^ mice with diseases on syngeneic CD8 T cell proliferation in co-culture assays. CD8 T cells were purified from naïve LMC mice, labeled with the cell proliferation dye eFluor 670, and stimulated with anti-CD3 or anti-CD3+anti-CD28 in the absence or presence of purified CD11b+Gr-1+ cells (at a 1:1 ratio to T cells) for 4 days. Gated populations in representative FACS histograms are proliferated CD8+ T cells with the labeled proliferation dye diluted. **(E)** Graphical results of the percentage of proliferated CD8 T cells obtained from 3 experiments. **(F–J)** Hyperexpansion of MDSCs in young adult M-*Traf3* ^-/-^ mice (2-4-month-old) as compared to age- and gender-matched LMC mice after repeated injections with heat-killed BCG. **(F)** Increased spleen weight of M-*Traf3* ^-/-^ mice at day 2 after the 3^rd^ BCG injection (n=10/group). **(G)** Representative FACS profiles of splenocytes stained for B220, CD3, CD11b, Gr1, Ly6C, or Ly6G. **(H)** Graphical results of the percentage and number of splenic CD11b+Gr1+ cells, G-MDSCs (CD11b+Ly6G+Ly6C^int^), and M-MDSCs (CD11b+Ly6G-Ly6C^hi^) (n=10/group). **(I)** Kinetic change of the percentage and number of splenic MDSCs at different time points after BCG injections (n=6/group). **(J)** Comparable suppressive activity of LMC and M-*Traf3* ^-/-^ MDSCs purified from mouse spleens at day 2 after the 3^rd^ BCG injection on syngeneic CD8 T cell proliferation in co-culture assays. Graphical results of the percentage of proliferated CD8 T cells were obtained from 3 experiments. Graphs **(E, F, H–J)** depict the mean ± SEM (n.s., *p* > 0.05; *p < 0.05; **p < 0.01; ***p < 0.001). The *p* values were determined by *t* test **(A, F, H)** or ANOVA **(E, I, J)**.

We recently reported that aging M-*Traf3* ^-/-^ mice (15-22-month-old) exhibit a spontaneous disease phenotype, characterized by chronic inflammation and tumor development that affect multiple organs (27). Interestingly, we noticed that such aging M-*Traf3* ^-/-^ mice with spontaneous chronic inflammation or tumor development are consistently associated with a remarkable expansion of CD11b+Gr1+ myeloid cells in the spleen, which could be neutrophils or MDSCs (27, 28) (**Figures 1A**, **S1A**). The expanded CD11b+Gr1+ cells include two subsets, Ly6G+Ly6C^int^ and Ly6G-Ly6C^hi^ cells (**Figure 1B**). To distinguish these cells from mature neutrophils, we determined their nuclear morphology as ring-shaped or oval-shaped (**Figure 1C**), which are characteristic of granulocytic MDSC (G-MDSC) and monocytic MDSC (M-MDSC), respectively. Furthermore, splenic CD11b+Gr1+ cells purified from aging M-*Traf3* ^-/-^ mice (15-22-month-old) with spontaneous diseases exhibited potent immunosuppressive activity on syngeneic CD8 T cell proliferation in co-culture assays (**Figures 1D, E**). Thus, the CD11b+Gr1+ cells aberrantly expanded in aging M-*Traf3* ^-/-^ mice with spontaneous chronic inflammation or tumors are MDSCs.

Given the prominent roles of MDSCs in chronic inflammation and immunosuppression as well as their critical importance in cancer immunotherapies (2–8), the above evidence led us to test the hypothesis that TRAF3 expressed in myeloid cells can negatively regulate MDSC expansion during chronic inflammation. To test this, we employed an induction model of chronic inflammation in young adult (2-4-month-old) mice using repeated injections of heat-killed BCG (31). After 3 BCG injections, young adult M-*Traf3* ^-/-^ mice exhibited a significant increase in spleen weights and a hyperexpansion of splenic MDSCs as compared to that observed in gender- and age-matched littermate control (LMC) mice (**Figures 1F–H**). In contrast, the frequencies of splenic B cells and T cells were decreased in M-*Traf3* ^-/-^ mice (**Figure S1B**). This appears to be an indirect result of the hyperexpansion of MDSCs, because the numbers of splenic B cells and T cells in M-*Traf3* ^-/-^ mice were not decreased as compared to those observed in LMC mice (**Figure S1B**). We next compared the kinetics of MDSC expansion and contraction between the two genotypes of mice (**Figures 1I**, **S1C, D**). Our results demonstrated that *Traf3* deficiency robustly increased the magnitude, but did not affect the duration, of MDSC expansion in this model of chronic inflammation (**Figure 1I**). We further investigated if TRAF3 regulates the immunosuppressive capacity of MDSCs. We purified MDSCs from the spleens of young adult M-*Traf3* ^-/-^ and LMC mice at day 2 after the 3^rd^ BCG injection for co-culture experiments with syngeneic CD8 T cells. Our data showed that M-*Traf3* ^-/-^ and LMC MDSCs were equally immunosuppressive on CD8 T cell proliferation (**Figures 1J**, **S1E**). Together, our results demonstrate that TRAF3 is a novel regulator of MDSC expansion but does not affect the constitutive immunosuppressive activity of MDSCs.

### Impaired tumor immunity in M-*Traf3* ^-/-^ mice with chronic inflammation

**Figure 2:**
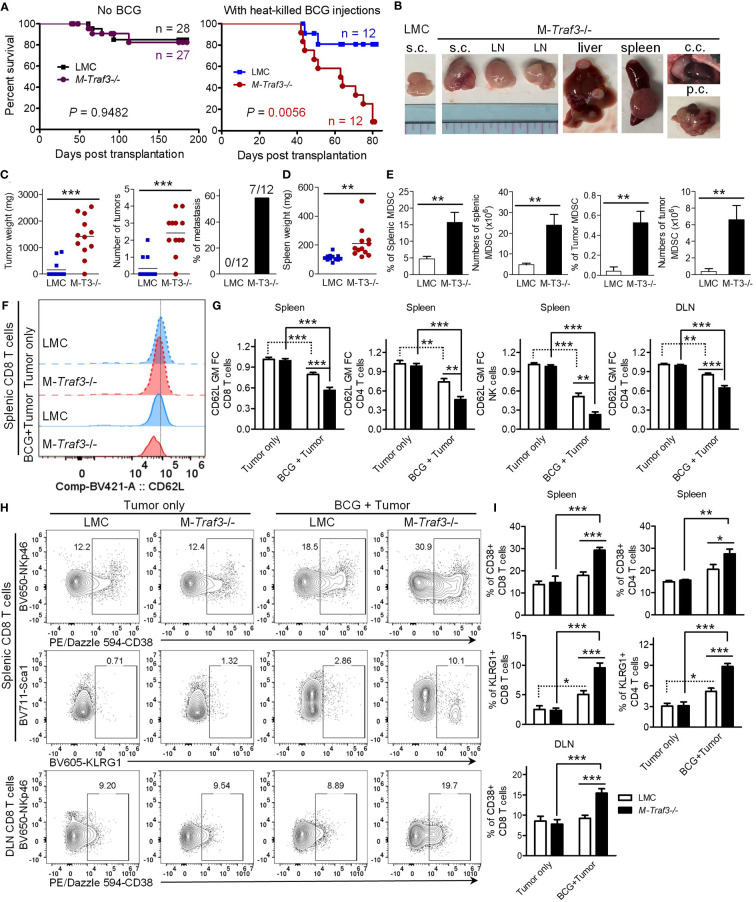
Young adult M-*Traf3* ^-/-^ mice that received repeated injections of heat-killed BCG failed to control tumor growth and metastasis. Gender-matched, young adult naïve LMC and M-*Traf3* ^-/-^ mice (2-4-month-old) or mice that received 3x BCG injections were transplanted *s.c.* with 5x10^6^ cells of the 291-6 and 291-7 DLBCL cell lines. **(A–E)** End-point analyses of the transplanted mice. **(A)** Survival curves of mice after tumor transplantation as analyzed by the Kaplan-Meyer method. **(B)** Representative images of lymphomas in BCG-treated mice dissected from the transplanted site (*s*.*c*.), LN, liver, spleen, chest cavity (c.c.), or peritoneal cavity (p.c.). **(C)** Graphical results of the tumor weight, the number of tumors, and the incidence of metastasis in transplanted LMC and M-*Traf3* ^-/-^ (M-T3-/-) mice that received repeated injections of heat-killed BCG (n=12/group). **(D)** Spleen weight of the transplanted and BCG-treated mice (n=12/group). **(E)** The percentage and number of MDSCs in the spleen and TME of the transplanted and BCG-treated mice. **(F–I)** FACS analyses of the transplanted mice at day 7 post transplantation. **(F)** Representative FACS histogram overlay comparing the expression level of CD62L on gated splenic CD8 T cells in transplanted LMC and M-*Traf3* ^-/-^ mice without (Tumor only) or with BCG treatment (BCG + Tumor). **(G)** Graphical results of the fold of change (FC) of the geometric mean (GM) of CD62L staining intensity on gated CD8 T cells, CD4 T cells, or NK cells in the spleen or DLN of transplanted mice (n=8/group). **(H)** Representative FACS profiles of CD38, NKp46, KLRG1, or Sca1 staining on CD8 T cells of the spleen or DLN of transplanted mice. Gated populations are CD38+ or KLRG1+ subsets. **(I)** Graphical results of the percentage of CD38+ or KLRG1+ subsets of CD8+ or CD4+ T cells in the spleen or DLN of transplanted mice (n=6/group). Graphs **(E, G, I)** depict the mean ± SEM. The *p* values were determined by the Mantel-Cox log-rank test **(A)**, *t* test **(C–E)**, or ANOVA **(G, I)**: *p < 0.05; **p < 0.01; ***p < 0.001.

MDSCs can promote tumor growth and facilitate tumor metastasis, while suppressing tumor immunity in various cancers (4–9). We reasoned that MDSC hyperexpansion would accelerate tumor progression and compromise anti-tumor immunity in M-*Traf3* ^-/-^ mice with chronic inflammation. To test this possibility, we exploited a new tumor transplantation model using the cell lines 291-6 and 291-7 that we generated from primary diffuse large B-cell lymphomas (DLBCL) spontaneously developed in two aging M-*Traf3* ^-/-^ mice. Naïve young adult (2-4-month-old) LMC and M-*Traf3* ^-/-^ mice were able to reject the transplanted DLBCL cells (**Figure 2A**). However, in the presence of BCG-induced chronic inflammation, the transplanted DLBCL cells developed into tumors in 25% of LMC mice and almost all (91.7%) of the examined M-*Traf3* ^-/-^ mice, leading to significantly shortened survival of the knockout recipients (**Figures 2A–C**). BCG-treated M-*Traf3* ^-/-^ mice exhibited not only an aggravated growth of the transplanted lymphomas but also frequent metastasis to other organs, which was associated with a significant increase in the spleen weight and a drastically enhanced MDSC expansion in the spleen, draining lymph node (DLN), and tumor microenvironment (TME) (**Figures 2B–E**, **S2, S3A**). Thus, these data demonstrate that in the presence of chronic inflammation, *Traf3* deficiency in myeloid cells is sufficient to confer the susceptibility to tumor growth and metastasis.

It has been shown that MDSCs can potently suppress tumor immunity by directly acting on CD8 and CD4 T lymphocytes as well as NK cells (2, 8). To understand how MDSC hyperexpansion in M-*Traf3* ^-/-^ mice may impair tumor immunity, we first examined the expression level of the immunosuppressive ligand PD-L1 on MDSCs in our mouse models. Interestingly, the expression level of PD-L1 was up-regulated on MDSCs in the DLN and further up-regulated in the TME (**Figure S3B**). This is consistent with previous reports that tumor-infiltrating MDSCs express higher level of PD-L1 than splenic or blood MDSCs in other cancer models (35, 36). However, we did not detect any significant difference in PD-L1 expression between the two genotypes (LMC and M-*Traf3* ^-/-^) of MDSCs harvested from the same anatomical location (**Figure S3B**). Notably, MDSCs of the TME expressed a markedly higher level of PD-L1 than the transplanted lymphoma cells (**Figure S3C**). Although we did not test whether PD-L1 functionally drives the immunosuppressive effect of MDSCs in this study, it has been well-established that PD-L1/PD-1 signaling is an important component of immunosuppression in the TME (37). In this context, the presence of increased frequency and number of MDSCs expressing high levels of PD-L1 in the TME of M-*Traf3* ^-/-^ mice would contribute to deeper inhibition of lymphocyte function, leading to enhanced suppression of tumor immunity. We thus investigated the effects of BCG-induced MDSC expansion on the phenotype of T lymphocytes and NK cells after tumor transplantation. We observed significantly down-regulated expression levels of the adhesion molecule CD62L (L-selectin), a known target of MDSCs (38), on CD8 T cells, CD4 T cells, and NK cells in the spleen and DLN of BCG-treated mice at day 7 post tumor transplantation, which were further exacerbated by *Traf3* deficiency in myeloid cells (**Figures 2F, G**, **S4A, B**). Interestingly, the immunosuppressive ectoenzyme CD38 (39–41) and the co-inhibitory receptor KLRG1 (42, 43) were induced on strikingly increased frequencies of CD8 T cells, CD4 T cells, and NK cells in the spleen and DLN of BCG-treated M-*Traf3* ^-/-^ mice after transplantation (**Figures 2H, I**, **S4C, D**). CD38^hi^ subsets of CD8 T cells, CD4 T cells, and NK cells have been shown to be immunosuppressive (44–46), while KLRG1+ T cell subsets lack proliferative capacity and express high levels of FoxP3 (47–49). Thus, our results suggest that the altered phenotype of T cells and NK cells in lymphoid organs of M-*Traf3* ^-/-^ mice with chronic inflammation appears to be immunosuppressive or senescent, favoring tumor growth and metastasis. Together, our findings reveal an interesting “trans-cell” tumor suppressive function of TRAF3 as demonstrated by the evidence that TRAF3 expressed in myeloid cells can suppress the development or progression of tumors derived from another cell type such as B lymphocytes.

### Biased expansion of M-*Traf3^-/-^*-derived MDSCs in mixed BM chimeras

**Figure 3:**
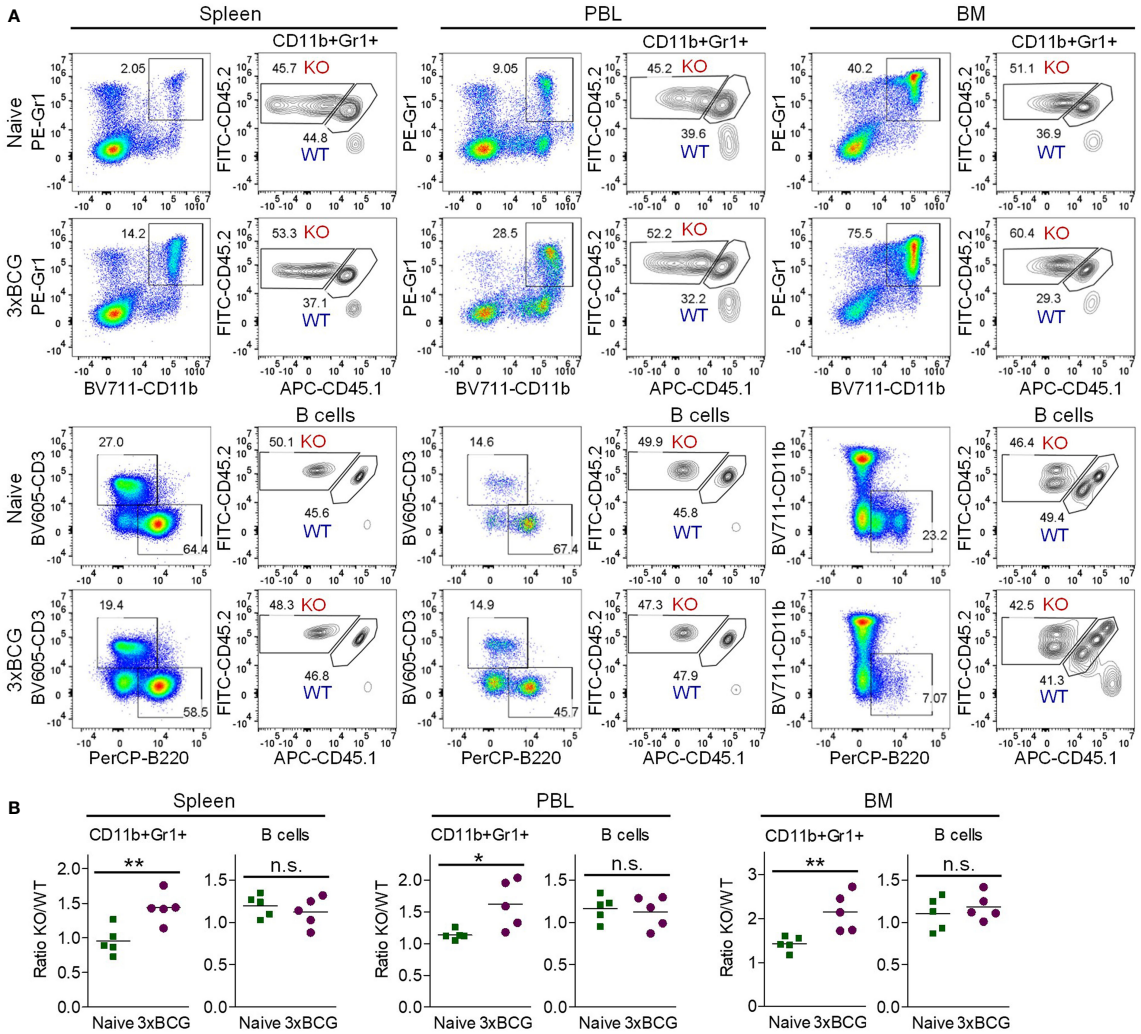
Increased expansion of M-*Traf3* ^-/-^ MDSCs in mixed BM chimeras after repeated injections of heat-killed BCG. BM cells derived from gender-matched, young adult WT (CD45.1/CD45.2) and M-*Traf3* ^-/-^ (CD45.2) mice were mixed at a 1:1 ratio and adoptively transferred into lethally irradiated C57.SJL (CD45.1) recipient mice. At 8 weeks after reconstitution, a cohort of mixed BM chimeras were subjected to repeated *s.c.* injections of heat-killed BCG. On day 2 after the 3^rd^ BCG injection (3xBCG), the BCG-treated and naïve chimeras that were generated from the same experiments were harvested and analyzed together. **(A)** Representative FACS profiles of M-*Traf3* ^-/-^ (KO, CD45.2+CD45.1-)-derived and WT (CD45.1+CD45.2+)-derived CD11b+Gr1+ cells and B cells (B220+CD3-CD11b- gated) in the spleen, PBL and BM of naïve *versus* BCG-treated mixed BM chimeras. **(B)** Graphical results comparing the ratio of KO/WT of gated CD11b+Gr1+ cells or B cells (B220+CD3-CD11b-) in the spleen, PBL, and BM of naïve *versus* BCG-treated mixed BM chimeras (n=5/group; n.s., *p* > 0.05; *p < 0.05; **p < 0.01 as determined by *t* test).

In light of the clinical importance of MDSCs in cancer immunotherapies (2–4), we next sought to elucidate how TRAF3 inhibits MDSC expansion. To unambiguously distinguish whether TRAF3 inhibits MDSC expansion by acting inside MDSCs and/or their progenitor cells (cell-intrinsic mechanism) or by affecting environmental factors released from non-MDSC myeloid cells (cell-extrinsic mechanism), we generated mixed BM chimeras. At 8 weeks after reconstitution, we verified the chimerism in peripheral blood cells (PBL). A cohort of BM chimeras were treated with repeated injections of heat-killed BCG to induce MDSC expansion. BCG-treated and naïve BM chimeras that were generated in the same experiments were sacrificed together for comparison. We found that at day 2 after the 3^rd^ BCG injection, the ratio of M-*Traf3* ^-/-^/WT CD11b+Gr1+ cells was significantly increased at approximately 1.5-fold in the spleen, PBL, and BM of the BCG-treated mixed BM chimeras as compared to that in the naïve BM chimeras (**Figures 3A, B**). In contrast, the ratio of M-*Traf3* ^-/-^/WT B cells and T cells were not significantly changed by BCG treatment (**Figures 3A, B**, **S5A, B**). Notably, the 1.5-fold increment of M-*Traf3* ^-/-^/WT CD11b+Gr1+ cells detected in BCG-treated mixed BM chimeras (**Figure 3B**) is much lower than the 3.95-fold increase of splenic MDSC number observed in M-*Traf3* ^-/-^ mice after BCG treatment (**Figure 1H**), indicating that environmental factors present in M-*Traf3* ^-/-^ mice also contribute to MDSC hyperexpansion. Thus, our evidence demonstrates that TRAF3 inhibits MDSC expansion *via* both cell-intrinsic and cell-extrinsic mechanisms.

### G-CSF is a major driver of MDSC hyperexpansion in M-*Traf3* ^-/-^ mice

**Figure 4:**
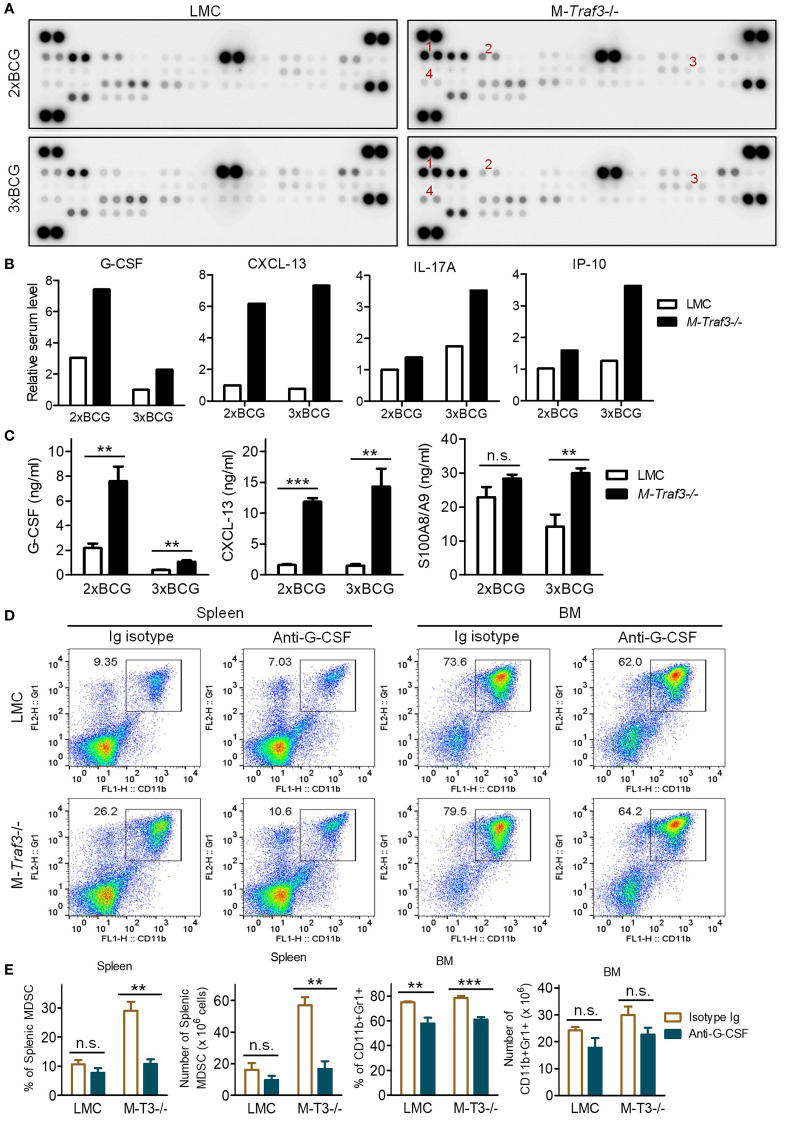
Elevated G-CSF was a major inflammatory mediator that drove MDSC hyperexpansion in BCG-treated M-*Traf3* ^-/-^ mice. **(A-C)** Sera were collected from gender-matched, young adult LMC and M-*Traf3* ^-/-^ mice (2-4-month-old) at day 2 after the 2^nd^ (2xBCG) or 3^rd^ (3xBCG) injection with heat-killed BCG. **(A)** Cytokine and chemokine protein array blots. Cytokines and chemokines in mouse sera were detected using the Mouse Cytokine Array Assay kit (R&D) following the manufacturer’s protocol. In each blot, the combined sera of 4 mice (50 μl serum/mouse) were used for the cytokine array assay. Red numeric labels indicate the spots that are obviously different between M-*Traf3* ^-/-^ and LMC mice: 1, CXCL-13; 2, G-CSF; 3, IL-17; 4, IP-10. **(B)** Quantification of cytokine and chemokine levels of mouse sera measured by the cytokine protein array analyses. Duplicate spots of each cytokine or chemokine on the blots in **(A)** were quantitated using a low-light imaging system and the fold of change (mean) was presented graphically. The intensity of each cytokine or chemokine spot was relative to the mean of that of the corresponding LMC spots of 2xBCG. **(C)** Serum levels of G-CSF, CXCL-13, and S100A8/A9 measured by individual ELISA (n=6/group). **(D, E)** *In vivo* neutralization of G-CSF in gender-matched, young adult LMC and M-*Traf3* ^-/-^ mice. Beginning at day 1 after the 1^st^ BCG injection, mice were injected *i.p.* with 25 µg of anti-G-CSF neutralizing antibody or Ig isotype control thrice weekly for 2 weeks. Mice were dissected on day 2 after the 3^rd^ BCG injection. **(D)** Representative FACS profiles of splenocytes and BM cells stained for CD11b and Gr1. **(E)** Graphical results of the percentage and number of CD11b+Gr1+ MDSCs in the spleen and BM obtained from 3 experiments. Graphs **(C, E)** depict the mean ± SEM (n.s., *p* > 0.05; **p < 0.01; ***p < 0.001 as determined by ANOVA).

To elucidate the cell-extrinsic mechanism underlying TRAF3-mediated inhibition of MDSC expansion, we aimed to identify the environmental factors important for MDSC expansion that were aberrantly elevated in M-*Traf3* ^-/-^ mice. Using a Cytokine Protein Array and standard ELISAs, we measured the serum levels of 40 cytokines/chemokines and 2 additional inflammatory mediators (HMGB1 and S100A8/A9) pivotal for MDSC expansion (**Figures 4A–C**, **S5C**). Of the 42 examined mediators, only G-CSF and CXCL-13 displayed a drastic elevation both at a relatively early time point in the BCG model (**Figures 4A–C**) and in aging M-*Traf3* ^-/-^ mice with spontaneous chronic inflammation and tumors (27), suggesting their potential importance in TRAF3-mediated regulation of MDSC expansion.

G-CSF is known as a principal inflammatory mediator that drives MDSC mobilization and expansion (8). G-CSF also induces extramedullary proliferation of MDSCs in the spleen (8). Indeed, we observed increased percentage and number of proliferating MDSCs in the spleen of M-*Traf3* ^-/-^ mice after BCG treatment as demonstrated by *in vivo* 5-bromo-2′-deoxyuridine (BrdU) labelling (**Figures S6A, B**), suggesting extramedullary proliferation of MDSCs in BCG-treated M-*Traf3* ^-/-^ mice. Interestingly, CXCL-13 was recently shown to induce the migration of MDSCs (50). To assess if elevated serum levels of G-CSF and CXCL-13 play a causal role for MDSC hyperexpansion in M-*Traf3* ^-/-^ mice, we performed *in vivo* neutralization experiments. We showed that neutralization of G-CSF, but not CXCL-13, effectively inhibited splenic MDSC hyperexpansion in M-*Traf3* ^-/-^ mice after BCG treatment (**Figures 4D, E**, **S6C, D, S7**). Neutralization of G-CSF also modestly reduced the percentage of CD11b+Gr1+ cells in the BM of both LMC and M-*Traf3* ^-/-^ mice (**Figures 4D, E**). More specifically within the gated CD11b+ population, neutralization of G-CSF decreased the percentage and number of Ly6G+Ly6C^int^ G-MDSCs, but not Ly6G-Ly6C^hi^ M-MDSCs, in the spleen and BM of BCG-treated M-*Traf3* ^-/-^ mice (**Figures S6C, D**). Together, our results reveal that elevated serum G-CSF is a major mediator of MDSC hyperexpansion in M-*Traf3* ^-/-^ mice.

### Increased G-CSF-producing inflammatory monocytes and macrophages in M-*Traf3* ^-/-^ mice

**Figure 5:**
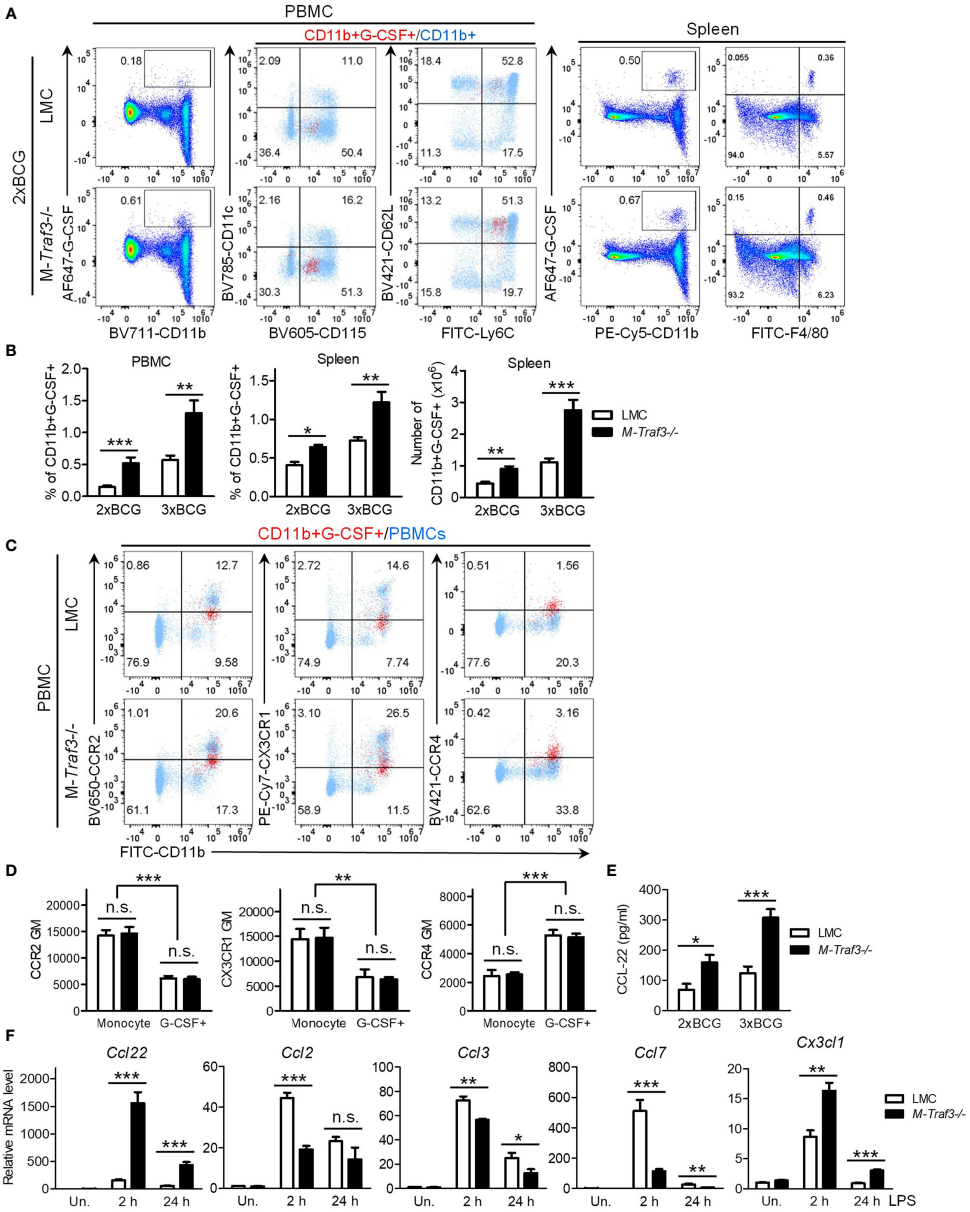
Increased percentage and number of G-CSF-producing myeloid cells in M-*Traf3* ^-/-^ mice after repeated injections with heat-killed BCG. **(A-D)** PBMCs and splenocytes were harvested from gender-matched, young adult LMC and M-*Traf3* ^-/-^ mice (2-4-month-old) at day 1 after the 2^nd^ (2xBCG) or 3^rd^ (3xBCG) injection with heat-killed BCG. **(A)** Representative FACS profiles of PBMCs and splenocytes stained for G-CSF, CD11b, or F4/80. For PBMCs, CD11b+G-CSF+ gated cells (red) were overlaid on top of CD11b+ gated cells (light blue) to compare their expression of CD115, CD11c, Ly6C, and CD62L. **(B)** Graphical results of the percentage and number of CD11b+G-CSF+ myeloid cells in PBMCs and splenocytes of LMC and M-*Traf3* ^-/-^ mice at day 1 after the 2^nd^ or 3^rd^ BCG injection (n=6/group). **(C)** Representative FACS profiles showing CD11b+G-CSF+ gated PBMCs (red) overlaid on top of ungated PBMCs (light blue) to compare their expression of CCR2, CX3CR1, and CCR4. PBMCs shown were harvested at day 1 after the 3^rd^ BCG injection. **(D)** Graphical results of the geometric mean (GM) of the staining intensity of CCR2, CX3CR1, and CCR4 on CD11b+CD115+Ly6C+ gated general monocytes (Monocyte) *versus* G-CSF+ monocytes (G-CSF+) in PBMCs harvested at day 1 after the 3^rd^ BCG injection (n=4/group). **(E)** Serum levels of CCL-22 measured by ELISA (n=6/group). Mouse sera were collected from young adult LMC and M-*Traf3* ^-/-^ mice at day 2 after the 2^nd^ or 3^rd^ BCG injection. **(F)** LPS-induced mRNA expression of *Ccl22*, *Ccl2*, *Ccl3*, *Ccl7*, and *Cx3cl1* in cultured BMDMs. BMDMs were prepared from gender-matched, young adult LMC and M-*Traf3* ^-/-^ mice. Cells were serum-starved for 2 h and then cultured in the absence (Un.) or presence of 100 ng/ml LPS for 2 or 24h. Quantitative real-time PCR of cDNA samples was performed using TaqMan assays. Relative mRNA levels were analyzed using the comparative Ct method. Results shown are from 3 experiments with duplicate reactions in each experiment. Graphs **(B-F)** depict the mean ± SEM (n.s., *p* > 0.05; *p < 0.05; **p < 0.01; ***p < 0.001 as determined by ANOVA).

To identify the cellular source that contributes to elevated serum G-CSF in M-*Traf3* ^-/-^ mice, we performed intracellular staining of peripheral blood mononuclear cells (PBMCs) and splenocytes for G-CSF-producing cells. We detected significantly increased percentages of G-CSF-producing CD11b+ myeloid cells in PBMCs and splenocytes of M-*Traf3* ^-/-^ mice after BCG injections (**Figures 5A, B**, **S8A**). In both genotypes of mice, the G-CSF-producing cells in PBMCs were mostly CD11b+CD115+CD11c-Ly6G-Ly6C+CD62L^hi^ inflammatory monocytes, whereas those in the spleen were mostly CD11b+F4/80+CD169+CD11c-MHC class II^lo^ macrophages (**Figures 5A**, **S8A**). In light of the recognition that TRAF3 is a negative regulator of both the noncanonical NF-κB (NF-κB2) pathway and c-Rel in macrophages (27, 28, 51, 52), and the evidence indicating that *Csf3* (encoding G-CSF) is a direct target gene of NF-κB (53, 54), one would predict that M-*Traf3* ^-/-^ macrophages, with elevated NF-κB2 p52/RelB and c-Rel, would produce a higher level of G-CSF per cell than LMC macrophages. However, we did not detect a significant difference in G-CSF production on a per cell basis between the two genotypes (LMC and M-*Traf3* ^-/-^) of gated CD11b+G-CSF+ monocytes and macrophages (**Figures 5A**, **S8A, B**), suggesting that TRAF3 does not directly inhibit G-CSF production in inflammatory monocytes and macrophages. Although the mechanism underlying this unexpected phenomenon remains to be determined, it is possible that NF-κB2 p52, RelB, and c-Rel are unable to bind to the promoter of the *Csf3* gene. Indeed, to date, direct binding with the *Csf3* promoter has only been demonstrated for p65 RelA or p50/p65 dimer in the literature (54, 55). Taken together, the above results indicate that elevated serum G-CSF in M-*Traf3* ^-/-^ mice is attributable to the increased frequencies and numbers of G-CSF-producing inflammatory monocytes and macrophages.

To understand why an increased frequency of G-CSF-producing inflammatory monocytes were present in PBMCs of M-*Traf3* ^-/-^ mice, we first tested the possibility that an increased proportion of M-*Traf3* ^-/-^ monocytes are induced to produce G-CSF in response to bacterial ligands. However, our data obtained from *in vitro* BM culture experiments negated this possibility. As shown in **Figures S9A, B**, LMC and M-*Traf3* ^-/-^ BM-derived monocytes, but not neutrophils, were equivalently induced to produce G-CSF following stimulation with the myeloid growth factor GM-CSF and bacterial lipopolysaccharides (LPS, a TLR4 agonist). We next tested an alternative possibility that TRAF3 inhibits the chemotaxis and trafficking of G-CSF-producing monocytes from the BM to blood circulation by down-regulating the expression of key chemokine receptors important for monocyte migration. For this purpose, we examined the expression levels of the following chemokine receptors: CCR2 - the primary receptor for monocyte emigration from the BM (56), CX3CR1 - the receptor important for monocyte recruitment to splenic sites (56), and CCR4 - a receptor up-regulated in certain subsets of inflammatory monocytes and monomyeloid precursors (57–60). Our results showed a robust up-regulation of CCR4 but down-regulation of CCR2 and CX3CR1 on BM-derived monocytes after stimulation with GM-CSF and LPS (**Figures S9C, D**). Interestingly, *in vivo* G-CSF-producing monocytes were also CCR2^lo^CX3CR1^lo^CCR4+, while general monocytes in PBMCs were CCR2^hi^CX3CR1^hi^CCR4- in BCG-treated mice (**Figures 5C, D**). However, TRAF3 did not affect CCR4, CCR2, or CX3CR1 expression on G-CSF-producing monocytes (**Figures 5C, D**, **S9C, D**). Together, our results indicate that TRAF3 does not inhibit G-CSF production in inflammatory monocytes and does not affect the expression of chemokine receptors that are involved in the mobilization of G-CSF-producing monocytes.

### Elevated serum levels of the CCR4 ligand CCL22 in M-*Traf3* ^-/-^ mice

Based on our results, which show an increased frequency of G-CSF-producing monocytes in PBMCs of M-*Traf3* ^-/-^ mice but no change in the induction of G-CSF production in monocytes by bacterial ligands or in the expression of monocyte chemokine receptors, we hypothesized that TRAF3 may regulate the production of relevant chemokines responsible for recruiting G-CSF-producing inflammatory monocytes. Using the Cytokine Protein Array and standard ELISAs, we determined serum levels of 15 chemokines, including CCLs (CCL-1~5, 11, 12 and 17) and CXCLs (CXCL-1~2 and 9~13). Among these, CCL-2 and CCL-12 are two ligands for CCR2 and known as the principal chemotactic factors for the emigration of monocytes from the BM (56), while CCL-17 is a ligand for CCR4. We detected most of the examined chemokines but did not notice a difference between LMC and M-*Traf3* ^-/-^ mice except for the aforementioned CXCL-13 and IP-10 (CXCL-10) (**Figures 4A–C**). We then measured the serum levels of additional key monocyte chemoattractants: CX3CL1 (the CX3CR1 ligand) and CCL-22 (a ligand of CCR4). Serum CX3CL1 in all examined mice were below the detection limit of ELISA. Interestingly, the serum level of CCL-22 was remarkably elevated in M-*Traf3* ^-/-^ mice following BCG injections (**Figure 5E**), supporting an increased recruitment of CCR4+ G-CSF-producing monocytes to the circulation.

It has been reported that CCL-22 is mainly produced by macrophages and DCs (61). To explore how TRAF3 regulates the serum CCL-22 level during inflammation, we investigated whether TRAF3 can inhibit the transcriptional expression of *Ccl22* and other chemokine genes in BM-derived macrophages (BMDMs) on a per cell basis. We detected drastically augmented transcript levels of *Ccl22* in M-*Traf3* ^-/-^ BMDMs after treatment with LPS (**Figure 5F**). LPS-induced expression of *Cx3cl1* was also significantly up-regulated in M-*Traf3* ^-/-^ BMDMs (**Figure 5F**), although serum CX3CL1 was not detectable by ELISA. In contrast, LPS-induced expression levels of *Ccl2* and *Ccl7* (encoding two ligands of CCR2) as well as *Ccl3* (encoding a ligand for CCR1 and CCR5) were down-regulated in M-*Traf3* ^-/-^ BMDMs (**Figure 5F**). Thus, TRAF3 impacts TLR4-induced production of monocyte chemoattractants, most strikingly CCL-22, in macrophages on a per cell basis. In line with our data, previous evidence indicates that *Ccl22* and *Cx3cl1* are direct target genes of NF-κB (62–68). In particular, both NF-κB1 and NF-κB2 have been shown to be essential for the expression of CCL-22 (63–65). Thus, the constitutive activation of NF-κB2 and elevated c-Rel in *Traf3* ^-/-^ macrophages (27, 28, 52) likely contribute to the heightened expression of *Ccl22* observed in M-*Traf3* ^-/-^ BMDMs and the increased serum CCL-22 level detected in BCG-treated M-*Traf3* ^-/-^ mice (**Figures 5E, F**). Collectively, our findings support the model that during chronic inflammation, TRAF3 inhibits TLR4-induced CCL-22 production in macrophages to limit the trafficking of CCR4+ G-CSF-producing monocytes to the circulation and their subsequent migration to the spleen, thereby restraining G-CSF-driven MDSC expansion and extramedullary proliferation in the spleen *via* a cell-extrinsic mechanism.

### Accelerated GM-CSF-induced proliferation of M-*Traf3* ^-/-^ CD11b+Gr1+ BM cells

**Figure 6:**
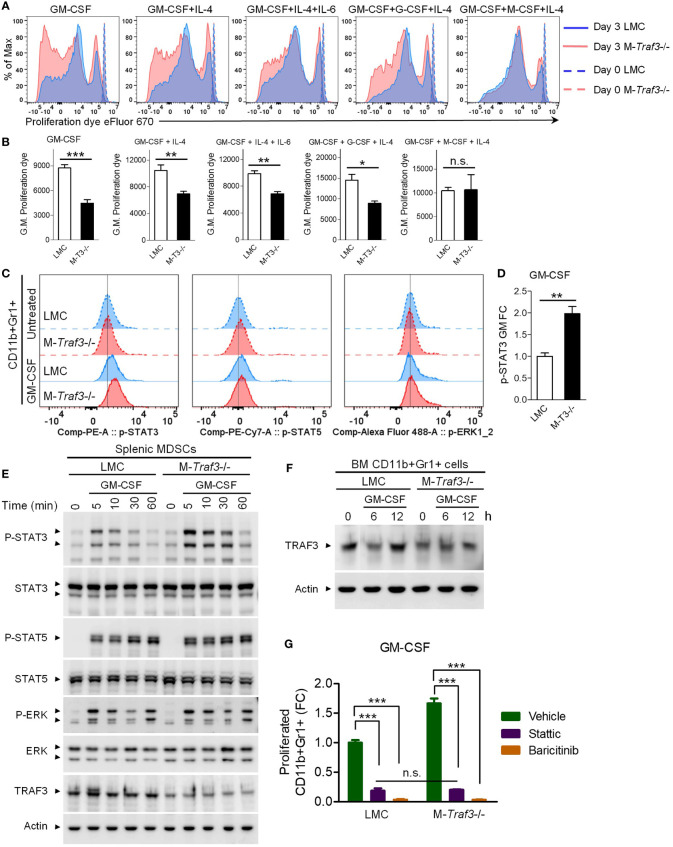
TRAF3 inhibited GM-CSF-induced proliferation and STAT3 phosphorylation in CD11b+Gr1+ cells. BM cells and splenic CD11b+Gr1+ cells were harvested from gender-matched, young adult LMC and M-*Traf3* ^-/-^ mice (2-4-month-old). **(A, B)** BM cells of naïve mice were labeled with the proliferation dye eFluor 670 and cultured in the presence of 20 ng/ml GM-CSF alone or 10 ng/ml GM-CSF in combination with 10 ng/ml of IL-4, IL-6, G-CSF, or M-CSF for 3 days. **(A)** Representative FACS histogram overlay comparing the dilution of the labeled proliferation dye in gated CD11b+Gr1+ BM cells after treatment for 3 days *versus* those prepared at day 0. **(B)** Graphical results of the geometric mean (G.M.) of the labeled proliferation dye intensity on gated CD11b+Gr1+ BM cells of LMC and M-*Traf3* ^-/-^ (M-T3-/-) mice (n=4/group). **(C-D)** BM cells of naïve mice were serum-starved for 2 h and treated without (Untreated) or with 20 ng/ml GM-CSF for 10 min. Phosphorylation of STAT3 (p-STAT3), p-STAT5, and p-ERK1/2 was subsequently analyzed by Phospho-Flow cytometry. **(C)** Representative FACS histogram overlay comparing p-STAT3, p-STAT5, and p-ERK1/2 staining on gated CD11b+Gr1+ BM cells. **(D)** Graphical results of the fold of change (FC) of the geometric mean (GM) of p-STAT3 staining intensity on gated CD11b+Gr1+ BM cells (n=4/group). **(E)** Splenic MDSCs were purified from mice at day 2 after the 3^rd^ injection with heat-killed BCG. Purified MDSCs were serum-starved for 2 h and then stimulated with 10 ng/ml GM-CSF for the indicated time (5-60 min). Total cellular protein lysates were immunoblotted for phosphorylated (P-) or total STAT3, STAT5, and ERK1/2 followed by TRAF3 and actin. **(F)** CD11b+Gr1+ cells were purified from the BM of naïve mice, serum-starved for 2 h, and then stimulated with 20 ng/ml GM-CSF for the indicated time (6 or 12 h). Total cellular protein lysates were immunoblotted for TRAF3 followed by actin. Blots shown are representative of 3 experiments. **(G)** BM cells of naïve mice were labeled with the proliferation dye eFluor 670 and cultured in the presence of 20 ng/ml GM-CSF, alone or in combination with 1 µM of Stattic or Baricitinib, for 3 days. Graphical results show the fold of change (FC) of proliferated CD11b+Gr1+ BM cells (n=4/group). Graphs **(B, D, G)** depict the mean ± SEM (n.s., *p* > 0.05; *p < 0.05; **p < 0.01; ***p < 0.001). The *p* values were determined by *t* test **(B, D)** or ANOVA **(G)**.

In addition to the above elucidated cell-extrinsic mechanism, the increased expansion of M-*Traf3* ^-/-^-derived MDSCs in mixed BM chimeras after BCG treatment (**Figure 3**) indicates that TRAF3 can also directly inhibit MDSC expansion by acting inside MDSCs and/or their progenitor cells through a cell-intrinsic mechanism. We further noticed that in naïve mixed BM chimeras, the ratio of M-*Traf3* ^-/-^/WT CD11b+Gr1+ cells in the BM was approximately 1.43, which was higher than that detected in the spleen (~0.95) (**Figure 3**). This led us to test if TRAF3 regulates CD11b+Gr1+ cell proliferation in response to growth factors present in the BM, including GM-CSF, G-CSF, M-CSF, IL-4, IL-6, alone or in combination. Interestingly, we found that among the stimuli examined, GM-CSF alone induced the most striking acceleration of proliferation in M-*Traf3* ^-/-^ CD11b+Gr1+ BM cells (**Figures 6A, B**, **S10A**), pointing to a critical role for TRAF3 in regulating GM-CSF-mediated MDSC proliferation. Given the vital importance of GM-CSF in MDSC expansion (7, 8), we chose to focus on TRAF3-mediated regulation of GM-CSF signaling in our further study.

### TRAF3 specifically inhibited GM-CSF-induced phosphorylation of STAT3

To elucidate how TRAF3 intrinsically inhibits GM-CSF-induced MDSC proliferation, we investigated the effects of *Traf3* deficiency on the early signaling events of GM-CSF in CD11b+Gr1+ cells. Our results from Phospho-Flow Cytometry revealed that GM-CSF-induced phosphorylation of STAT3, but not STAT5 or ERK1/2, was significantly enhanced in M-*Traf3* ^-/-^ CD11b+Gr1+ BM cells (**Figures 6C, D**). We also verified this finding using splenic MDSCs purified from BCG-treated mice. As demonstrated by Western blot analysis, GM-CSF-induced phosphorylation of STAT3, but not STAT5, ERK1/2, IκBα, RelA, Akt, GSK3β, or CREB, was markedly potentiated in splenic MDSCs purified from M-*Traf3* ^-/-^ mice (**Figures 6E**, **S10B**). Contrary to the constitutive activation of NF-κB2 and elevated c-Rel observed in *Traf3* ^-/-^ macrophages (27, 28, 52), we did not detect any alterations in NF-κB2 or NF-κB1 subunits in M-*Traf3* ^-/-^ MDSCs as compared to LMC MDSCs at basal levels or after GM-CSF stimulation (**Figure S10B**), suggesting that TRAF3 regulates GM-CSF signaling and GM-CSF-induced MDSC proliferation through NF-κB-**in**dependent mechanisms. Overall, our results revealed that TRAF3 specifically inhibits GM-CSF-induced STAT3 phosphorylation in both CD11b+Gr1+ BM cells derived from naïve mice and splenic MDSCs purified from BCG-treated mice.

It has been shown that the inhibitory role of TRAF3 on CD40 and LT-βR signaling is associated with TRAF3 degradation following receptor engagement by the corresponding ligands (11). We thus examined if GM-CSF induces TRAF3 degradation in CD11b+Gr1+ cells. We found that TRAF3 proteins were induced to undergo partial degradation in CD11b+Gr1+ BM cells purified from LMC mice at 6 h after GM-CSF stimulation (**Figure 6F**), corroborating our evidence that TRAF3 inhibited GM-CSF-induced STAT3 phosphorylation and cell proliferation (**Figures 6A–E**). Notably, we detected 30-50% residual TRAF3 proteins in both CD11b+Gr1+ BM cells and splenic MDSCs prepared from M-*Traf3* ^-/-^ mice (**Figures 6E, F**). However, we previously reported approximately 80-90% down-regulation of TRAF3 proteins in macrophages and neutrophils derived from M-*Traf3* ^-/-^ mice (27). The relatively higher extent of deletion of TRAF3 proteins in macrophages/neutrophils than in MDSCs is likely due to the higher expression level of the Cre recombinase driven by the lysozyme M promoter, which correlates with the differentiation stage of myeloid cells (69–71). We thus speculate that the cell-intrinsic effect of TRAF3 on MDSC expansion was underestimated with our mouse models and that had complete deletion of TRAF3 been possible, the cell-intrinsic effect of TRAF3 on MDSC expansion may have been more robust than that observed in the present study.

To determine if augmented STAT3 phosphorylation plays a causal role in accelerated GM-CSF-induced proliferation observed in M-*Traf3* ^-/-^ CD11b+Gr1+ cells, we examined the effects of a STAT3-specific inhibitor, Stattic, and an inhibitor of the upstream kinases JAK1/JAK2, Baricitinib (72, 73). Our results showed that GM-CSF-induced proliferation was substantially blocked by Stattic and almost completely abolished by Baricitinib in both LMC and M-*Traf3* ^-/-^ CD11b+Gr1+ BM cells (**Figure 6G**). Moreover, the enhanced GM-CSF-induced proliferation of M-*Traf3* ^-/-^ CD11b+Gr1+ BM cells was abrogated by treatment with Stattic or Baricitinib (**Figure 6G**), highlighting the importance of JAK-STAT3 activation in this process. Taken together, our results suggest that following GM-CSF stimulation, TRAF3 specifically inhibits STAT3 phosphorylation to hamper the proliferative response in CD11b+Gr1+ cells, thereby intrinsically restricting MDSC expansion.

### TRAF3 facilitated the association of STAT3 with the phosphatase PTP1B

**Figure 7:**
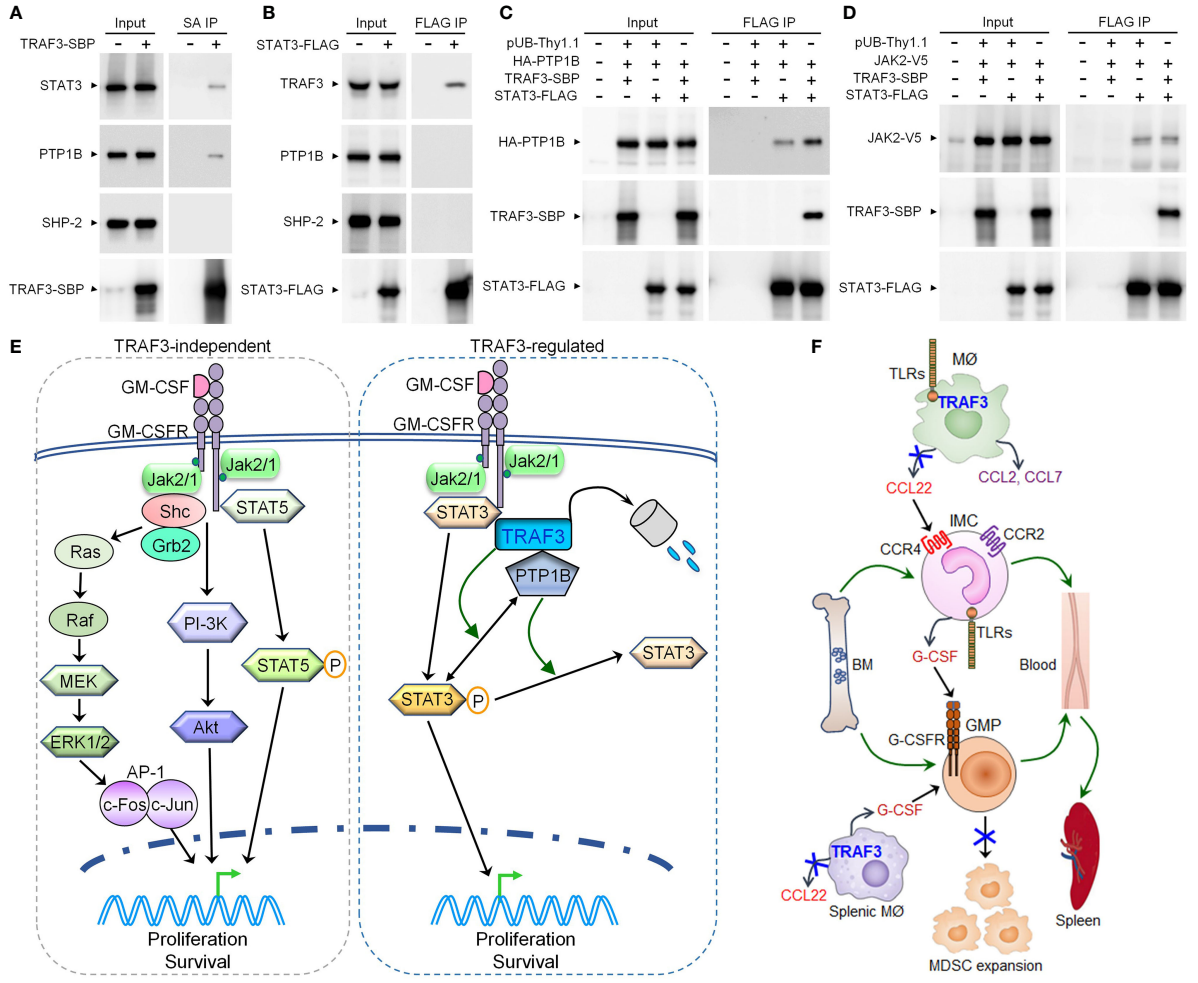
TRAF3 facilitated the association of STAT3 with PTP1B in co-transfected cells and pathway models elucidated in this study. **(A-D)** 293T cells were transfected with the expression vector of SBP-tagged TRAF3 (TRAF3-SBP), FLAG-tagged STAT3 (STAT3-FLAG), HA-tagged PTP1B (HA-PTP1B), V5-tagged JAK2 (JAK2-V5), or an empty vector pUB-Thy1.1, alone or in combination. Cells were harvested at day 2 post transfection. Total cellular proteins were immunoprecipitated with Streptavidin-Sepharose beads (SA) for TRAF3-SBP or anti-FLAG Affinity Gel for STAT3-FLAG. **(A)** Endogenous STAT3 and PTP1B, but not SHP-2, were co-immunoprecipitated with the transfected TRAF3-SBP. **(B)** Endogenous TRAF3, but not PTP1B or SHP-2, was co-immunoprecipitated with the transfected STAT3-FLAG. **(C)** Co-expression of TRAF3-SBP increased the association between STAT3-FLAG and HA-PTP1B in co-transfected 293T cells. **(D)** Co-expression of TRAF3-SBP did not affect the association between STAT3-FLAG and JAK2-V5 in co-transfected 293T cells. **(E)** A pathway model depicting TRAF3-mediated regulation of GM-CSF-STAT3 signaling inside MDSCs. GM-CSF induces partial degradation of TRAF3 in MDSCs. TRAF3 does not affect GM-CSF-induced activation of STAT5, ERK1/2, or Akt in MDSCs. In contrast, TRAF3 specifically inhibits GM-CSF-induced STAT3 phosphorylation by facilitating the recruitment of the phosphatase PTP1B, which in turn dephosphorylates STAT3 to restrict P-STAT3-dependent proliferation of MDSCs following GM-CSF stimulation. Therefore, TRAF3 expressed in MDSCs can inhibit GM-CSF-induced proliferation of these cells *via* a cell-intrinsic mechanism. **(F)** A schematic diagram illustrating TRAF3-mediated regulation of a novel TLR-CCL22-CCR4-G-CSF axis acting in inflammatory macrophages and monocytes. In inflammatory responses, TRAF3 inhibits TLR4-induced CCL22 production likely *via* an NF-κB2 and/or c-Rel-dependent mechanism in activated macrophages (MØ) of the skin and other tissues (including the spleen). Decreased CCL22 levels in the blood and spleen compromise the trafficking of CCR4+ G-CSF-producing inflammatory monocytes (IMC) from the BM to the circulation and their subsequent migration to the spleen, thereby reducing G-CSF levels in the blood and spleen. Reduced G-CSF levels lead to limited mobilization of granulocyte-monocyte progenitor cells (GMP) from the BM to the circulation and their extramedullary proliferation in the spleen, as both processes are primarily induced by G-CSF. Thus, TRAF3 expressed in macrophages can restrain G-CSF-driven MDSC expansion during chronic inflammation *via* a cell-extrinsic mechanism.

We next aimed to explore how TRAF3 specifically inhibits GM-CSF-induced STAT3 phosphorylation. To address this, we first interrogated the potential interaction between TRAF3 and STAT3. We demonstrated that endogenous STAT3 was co-immunoprecipitated with SBP-tagged TRAF3 (TRAF3-SBP) and endogenous TRAF3 was similarly co-immunoprecipitated with FLAG-tagged STAT3 (STAT3-FLAG) in transfected 293T cells (**Figures 7A, B**). We next investigated potential interactions between TRAF3 and two phosphatases, PTP1B and SHP-2, which mediate dephosphorylation of STAT3 in myeloid cells (74–76). Interestingly, we detected the co-immunoprecipitation of endogenous PTP1B, but not SHP-2, with TRAF3-SBP in these experiments (**Figure 7A**). However, co-immunoprecipitation of neither endogenous PTP1B nor SHP-2 with STAT3-FLAG was detected in transfected 293T cells (**Figure 7B**). This prompted us to test the possibility that TRAF3 may facilitate the association between STAT3 and PTP1B. Indeed, co-expression of TRAF3-SBP promoted the association between STAT3-FLAG and HA-PTP1B in co-transfected 293T cells as shown by co-immunoprecipitation (**Figure 7C**). In contrast, co-expression of TRAF3-SBP did not affect the association between STAT3-FLAG and its upstream kinase JAK2-V5 in co-transfected cells (**Figure 7D**). Collectively, our results suggest that TRAF3 specifically inhibits GM-CSF-induced STAT3 phosphorylation at least partially by facilitating the recruitment of PTP1B, which in turn dephosphorylates STAT3 to restrain the proliferation of CD11b+Gr1+ cells in a cell-intrinsic manner.

## Discussion

MDSCs expand under pathological conditions and are recognized as a prime target for the prevention and treatment of human diseases involving chronic inflammation and immunosuppression (2, 3, 77, 78). Intensive efforts are currently directed at developing therapeutic strategies to inhibit the expansion of MDSCs, to deplete MDSCs, and to block the immunosuppressive activities of MDSCs for cancers, chronic inflammatory diseases, chronic infection, and sepsis (2, 3, 77, 78). Therapeutic administration of MDSCs induced *in vitro* and induction of MDSCs *in vivo* are also being developed for conditions where immune suppression is desired, such as organ or stem cell transplantation and autoimmune disorders (79–82). Identification of new regulators of MDSCs is thus crucial for such therapeutic manipulation of MDSCs. In this study, we identified TRAF3 as a novel suppressor of MDSC expansion that acts *via* both cell-intrinsic and cell-extrinsic mechanisms.

For the cell-intrinsic mechanism, we elucidated that TRAF3 restrains GM-CSF-induced proliferation of CD11b+Gr1+ cells by specifically inhibiting the phosphorylation of STAT3 (**Figure 7E**), a central transcription factor for MDSC expansion (2, 7). In contrast, the NF-κB2 and NF-κB1 pathways, as well as GM-CSF-induced activation of STAT5, ERK1/2, and Akt, are not affected by TRAF3 in CD11b+Gr1+ BM cells or splenic MDSCs, arguing against their importance in TRAF3-mediated regulation inside MDSCs. Our co-immunoprecipitation studies further demonstrated that TRAF3 can interact with STAT3 and can facilitate the association of STAT3 with the phosphatase PTP1B. In line with our findings, Zhang et al. reported that PTP1B inhibits MDSC expansion through decreasing the activities of STAT3 and JAK2 (74). Analogous to the phenotype of M-*Traf3* ^-/-^ mice, PTP1B-deficient mice also exhibit MDSC hyperexpansion in a chronic inflammation model of DSS-induced colitis (74). Thus, TRAF3 specifically inhibits GM-CSF-induced STAT3 activation to restrict the proliferation of MDSCs at least partially by facilitating the recruitment of PTP1B, which in turn dephosphorylates STAT3 (**Figure 7E**). Elucidation of the GM-CSF-STAT3-TRAF3-PTP1B signaling axis in MDSCs provides additional targetable points and avenues for therapeutic manipulation of MDSC expansion in relevant disease settings.

In pursuing the cell-extrinsic mechanism, we discovered a novel TLR4-TRAF3-CCL22-CCR4-G-CSF axis acting in inflammatory macrophages and monocytes that drives and sustains MDSC expansion during chronic inflammation (**Figure 7F**). The CCL22-CCR4 axis has been recognized as an immune checkpoint for T cell immunity, as CCL22 is an essential chemoattractant for Treg cells (83). CCL22 is elevated in many types of human cancers and elevated CCL22 is associated with poor prognosis in patients (83). Therefore, targeting the CCL22-CCR4 axis by the anti-CCR4 antibody mogamulizumab or the CCR4 inhibitor AZD2098 is being tested as a promising strategy of cancer immunotherapy (83, 84). We found that G-CSF-producing inflammatory monocytes express heightened levels of CCR4 than resting monocytes and that M-*Traf3* ^-/-^ macrophages produce remarkably increased levels of CCL22 than LMC macrophages in response to bacterial ligands. Corroborating our findings, although most normal human blood monocytes do not express detectable CCR4, its expression is up-regulated on inflammatory monocytes in patients with inflammatory diseases (57–59). *Ccr4* ^-/-^ mice exhibit decreased mortality in sepsis models associated with attenuated inflammation and reduced recruitment of macrophages to the peritoneal cavity (85, 86). Furthermore, recent evidence suggests that CCL22 also directly induces MDSC recruitment to the TME and metastatic sites *via* CCR4 (87). Together, these findings would extend the use of drugs targeting the CCL22-CCR4 axis from blocking Treg cells to also inhibiting the expansion and migration of MDSCs in chronic inflammation and cancers.

Although our study highlights the importance of the GM-CSF-STAT3 and CCL22-CCR4-G-CSF axes, our results could not exclude the possibility that additional cell-intrinsic and cell-extrinsic mechanisms may also contribute to TRAF3-mediated regulation of MDSC expansion. Moreover, although *Traf3* deficiency does not affect the constitutive immunosuppressive activity of MDSCs, it remains possible that in the presence of various stimuli, especially in the TME, the immunosuppressive function of MDSCs may be regulated by TRAF3 through impacting relevant receptor signaling. In this regard, increasing evidence indicates that a number of TRAF3-employing immune receptors, including multiple members of both the TNF-R and TLR families, can directly regulate the immunosuppressive and pro-inflammatory functions of MDSCs (31, 88–92). These possibilities represent significant and exciting areas for future investigation.

Interestingly, we demonstrated that MDSC hyperexpansion led to accelerated growth and metastasis of transplanted tumors in young adult M-*Traf3* ^-/-^ mice, implicating that MDSC expansion is not simply a consequence but plays a causal role in tumor progression in aging M-*Traf3* ^-/-^ mice with spontaneous tumor development (27). The impaired tumor immunity in young adult M-*Traf3* ^-/-^ mice with MDSC hyperexpansion was associated with an altered phenotype of CD8 T cells, CD4 T cells, and NK cells. This alteration included down-regulation of the known MDSC target molecule CD62L (38) and up-regulation of the immunomodulatory receptors CD38 and KLRG1, which were not previously linked to MDSC-mediated alteration of lymphocyte phenotype, thereby suggesting a novel mechanism of MDSC-mediated immune suppression. CD38, a receptor for the adhesion molecule CD31, was traditionally used as a T cell activation marker, but most recent data indicate an immunosuppressive role for CD38, acting as an ectoenzyme to convert extracellular NAD^+^ into immunosuppressive adenosine (ADPR and cADPR) (39–41). KLRG1 is a co-inhibitory receptor and has been postulated to be a marker of senescence (42, 43). Upon binding to its ligand E-cadherin, KLRG1 delivers inhibitory signals *via* the cytoplasmic immunoreceptor tyrosine-based inhibition motif (ITIM) to dampen the functional responses of T cells and NK cells (42, 43). Both CD38 and KLRG1 expression on T cells are increased in the TME and up-regulated in human tumor samples after therapies, potentially contributing to adaptive resistance (40, 43, 93). In corroboration with our findings, blockades of CD38 and KLRG1 are being developed as therapeutic strategies to promote tumor immunity and to ameliorate resistance to PD-1/PD-L1 checkpoint inhibitors and other cancer therapies (40, 41, 43, 48, 93). Thus, our findings would further broaden the scope of therapeutic applications for CD38- and KLRG1-blocking drugs to various diseases involving aberrant MDSC expansion.

In summary, our findings provide novel mechanistic insights into how TRAF3 functions as a critical immune checkpoint in myeloid cells to restrain MDSC expansion, thereby preventing overzealous inflammation and indirectly promoting anti-tumor immunity. This knowledge will inform future translational and clinical efforts aimed at developing novel immunotherapies to manipulate MDSCs. Pharmacological stabilization of TRAF3 or reconstitution of TRAF3 expression in myeloid cells by gene therapy could be novel therapeutic approaches to prevent or reduce MDSC expansion. Discovery of the GM-CSF-STAT3-TRAF3-PTP1B signaling axis in MDSCs and the TLR4-TRAF3-CCL22-CCR4-G-CSF axis in inflammatory macrophages and monocytes provides a framework for targeting these crucial pathways by developing new drugs or by repurposing available drugs specific for STAT3, PTP1B, CCR4, or CCL22. Furthermore, the novel link between MDSCs and up-regulated expression of CD38 and KLRG1 on T cells and NK cells identified in this study suggests a wider scope of applications for CD38- and KLRG1-blocking drugs in immunotherapies of cancers and inflammatory diseases. These new therapeutic strategies in combination with current therapies would help to improve patient outcome.

## Data availability statement

The original contributions presented in the study are included in the article/**Supplementary Material**. Further inquiries can be directed to the corresponding author.

## Ethics statement

The animal study was reviewed and approved by Rutgers University IACUC.

## Author contributions

PX, SZ, AL, and SO-R designed the experiments of this study. DS’A and PX designed the mixed BM chimera experiments. KL, DS’A, and LC provided guidance on lymphoma transplantation and T cell analyses. HY supervised the cytokine analyses and macrophage experiments. SZ performed most experiments of this study. AL established the BCG-induced chronic inflammation model, the DLBCL cell lines for transplantation, and the co-culture model of MDSCs and CD8 T cells, and also performed the *in vivo* BrdU labeling experiments, the cytokine array assay, and initial GM-CSF signaling experiments. JJ helped to perform the lymphoma transplantation, *in vivo* neutralization, and cultured BMDM experiments. SZ, AL, JJ, and PX analyzed the data and performed the statistical analyses. PX, SZ, and AL wrote the manuscript. All authors contributed to the article and approved the submitted version.
