# Supplementary material for: The adaptor protein TRAF3 is an immune checkpoint that inhibits myeloid-derived suppressor cell expansion
Source: Front Immunol. 2023 May 3;14:1167924. doi: 10.3389/fimmu.2023.1167924 (PMC10189059; doi:10.3389/fimmu.2023.1167924)
Supplement: Supplementary file 1 [file DataSheet_1.pdf]

Supplementary Figures

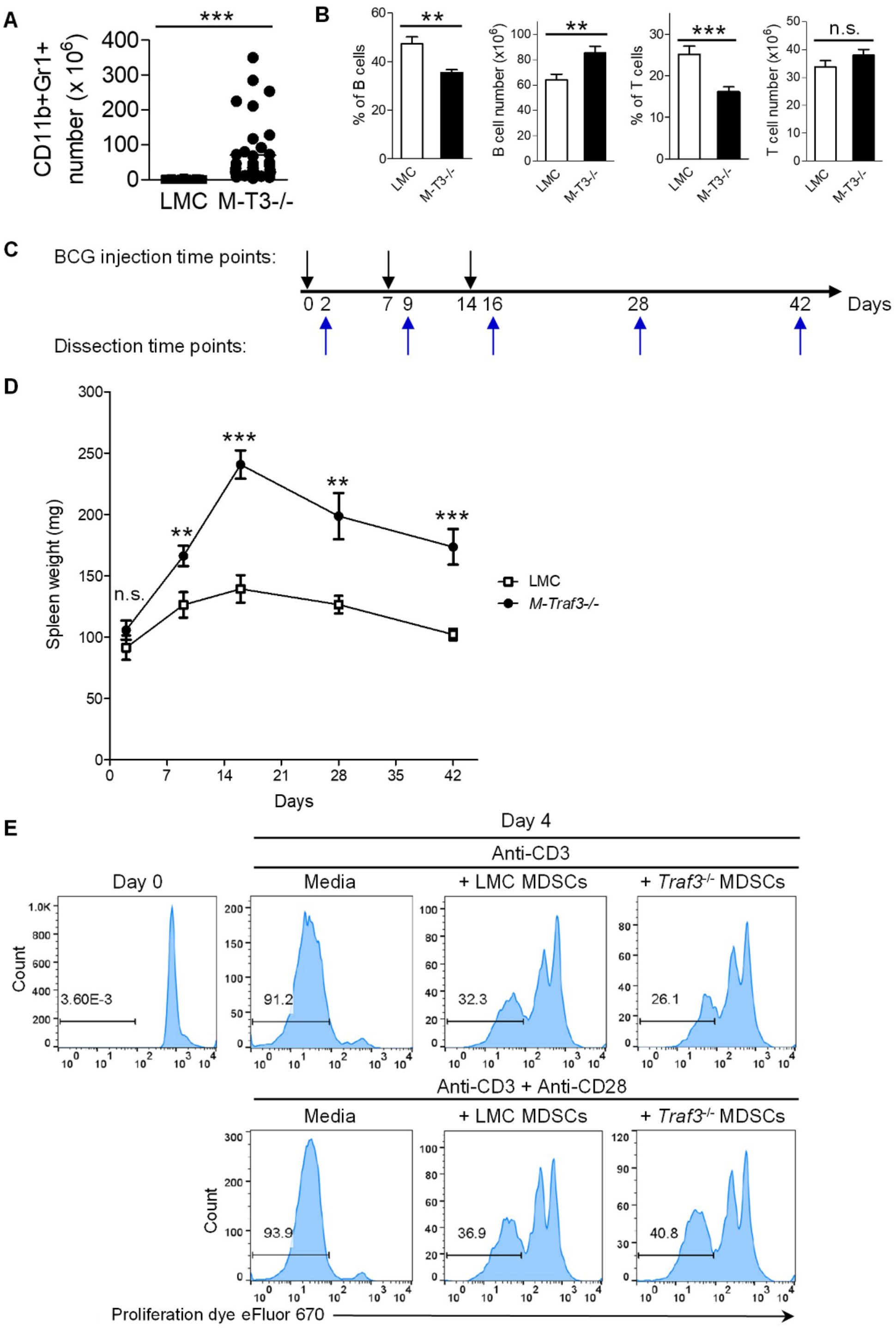

Figure S1. Analyses of splenic MDSCs in M-*Traf3*$^{-/-}$ mice. (**A**) Increased number of splenic CD11b+Gr1+ cells in aging M-*Traf3*$^{-/-}$ mice (15-22-month-old) with spontaneous chronic inflammation or tumors (n=36/group). (**B**) Graphical results of the percentage and number of splenic B cells (B220+CD3- gated) and T cells (CD3+B220- gated) in gender-matched, young adult LMC and M-*Traf3*$^{-/-}$ mice (2-4-month-old) at day 2 after the 3rd injection with heat-killed BCG (n=10/group). (**C**) Schematic diagram showing the schedule of BCG injections and dissection of mice for the kinetic study. (**D**) Kinetic change of the spleen weight of LMC and M-*Traf3*$^{-/-}$ mice at different time points after BCG injections (n=6/group). Graphs (B and D) depict the mean ± SEM (n.s., $p > 0.05$; **, $p < 0.01$; ***, $p < 0.001$). (**E**) Comparable suppressive activity of LMC and M-*Traf3*$^{-/-}$ MDSCs purified from mouse spleens at day 2 after the 3rd BCG injection on syngeneic CD8 T cell proliferation analyzed by co-culture experiments. CD8 T cells were purified from naïve LMC mice, labeled with the cell proliferation dye eFluor 670, and stimulated with anti-CD3 or anti-CD3+anti-CD28 in the absence or presence of purified LMC or M-*Traf3*$^{-/-}$ MDSCs (at a 1:1 ratio to T cells) for 4 days. Gated populations are proliferated CD8 T cells with the labeled proliferation dye diluted. FACS histograms shown are representative of 3 experiments. The p values were determined by t test (A and B) or ANOVA (D).

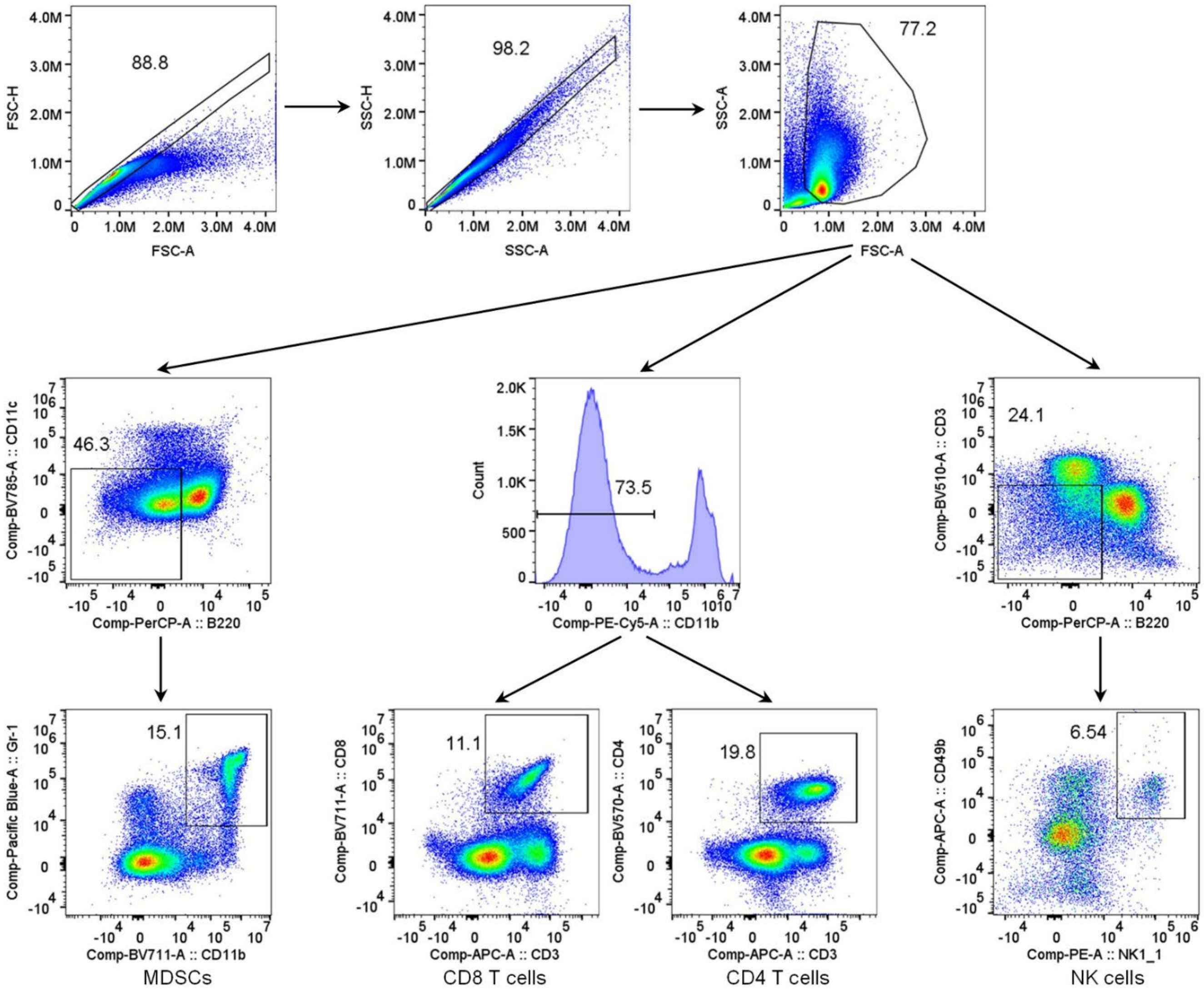

Figure S2. Representative gating strategy of FACS data shown in this manuscript. Cells were first gated according to FSC-A *versus* FSC-H, followed by SSC-A *versus* SSC-H and FSC-A *versus* SSC-A. The FSC and SSC gated single cells were subsequently gated according to the expression of different cell lineage markers, including B220, CD11c, CD11b, Gr1, CD3, CD8, CD4, NK1.1 and CD49b. MDSCs were gated as B220-CD11c-CD11b+Gr1+. CD8 T cells were CD11b-CD3+CD8+, while CD4 T cells were CD11b-CD3+CD4+. NK cells were gated as B220-CD3-NK1.1+CD49b+.

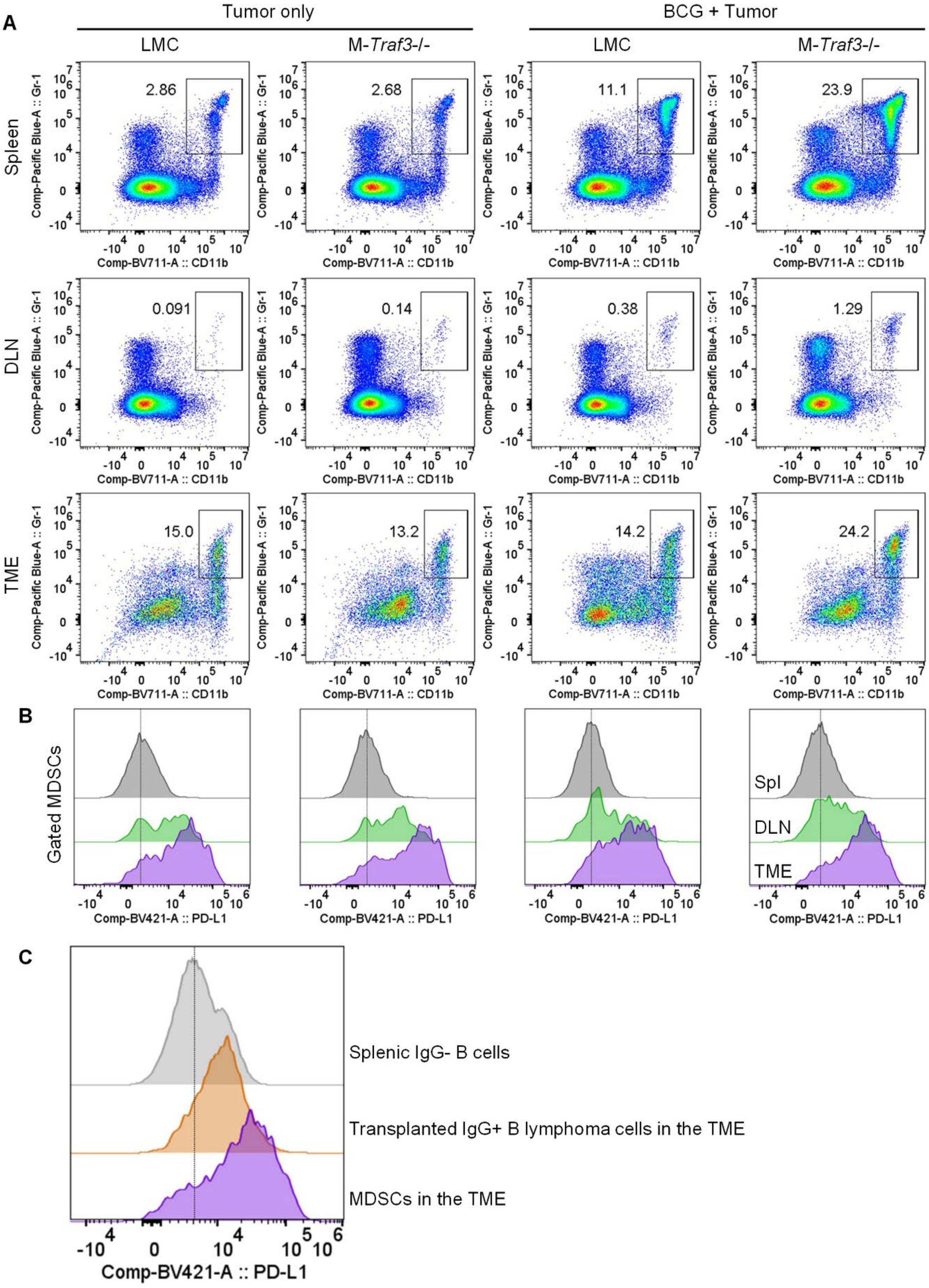

Figure S3. MDSC hyperexpansion in young adult M-*Traf3*$^{-/-}$ mice that received repeated injections of heat-killed BCG and tumor transplantation. Gender-matched, young adult naïve mice or mice that received 3x BCG injections were transplanted *s.c.* with 5x10^6 cells of the 291-6 and 291-7 B lymphoma (DLBCL) cell lines. FACS analyses were performed at day 7 post transplantation. (**A**) Representative FACS profiles of CD11b and Gr1 staining on mouse splenocytes, DLN cells, or cells dissociated from the transplanted Matrigel plug (tumor microenvironment, TME). Gated populations indicate the CD11b+Gr1+ MDSCs. (**B**) Representative FACS histogram overlay comparing PD-L1 expression on gated MDSCs in the spleen, DLN and TME. (**C**) Representative FACS histogram overlay comparing PD-L1 expression on gated splenic IgG-IgM+ B cells, transplanted IgG+IgM- B lymphoma cells, and MDSCs in the TME. Results shown are representative of 5 experiments.

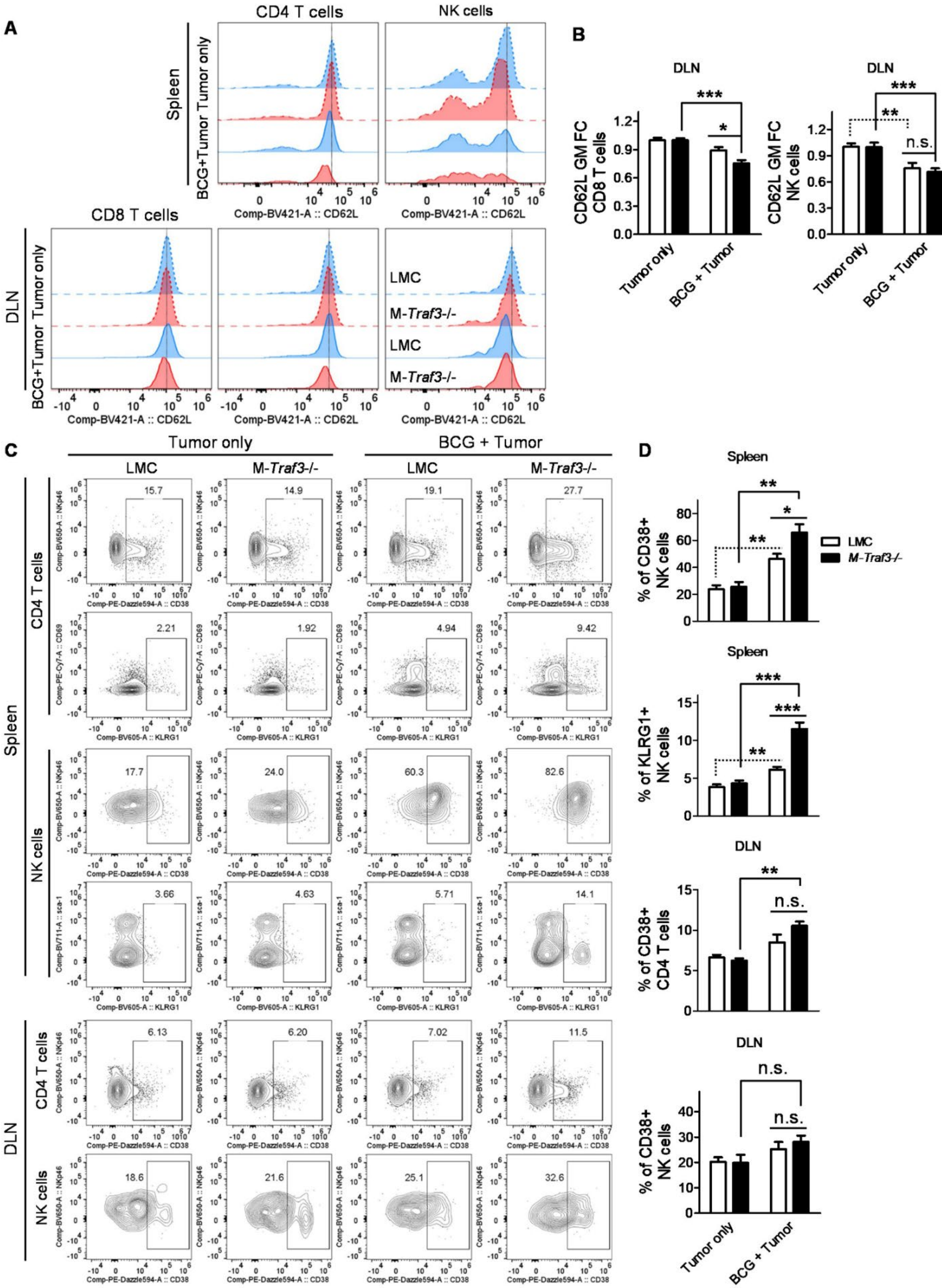

Figure S4. Altered T cell and NK cell phenotype in young adult M-*Traf3*$^{-/-}$ mice treated with heat-killed BCG and tumor transplantation. Gender-matched, young adult naïve mice or mice that received 3x BCG injections were transplanted *s.c.* with 5x10^{6} cells of the 291-6 and 291-7 B lymphoma cell lines. FACS analyses were performed at day 7 post transplantation. (**A**) Example FACS histogram overlay comparing CD62L expression on gated CD8 T cells, CD4 T cells and NK cells in the spleen and DLN. (**B**) Graphical results of the fold of change (FC) of the geometric mean (GM) of CD62L staining intensity on gated CD8 T cells and NK cells in the DLN (n=8/group). (**C**) Representative FACS profiles of CD38, NKp46, KLRG1 and CD69 staining on gated CD4 T cells and NK cells in the spleen or DLN. Gated populations are CD38+ or KLRG1+ subsets. (**D**) Graphical results of the percentage of CD38+ or KLRG1+ subsets of NK cells in the spleen as well as the percentage of CD38+ subsets of CD4 T cells and NK cells in the DLN (n=6/group). Graphs (B and D) depict the mean ± SEM (n.s., $p > 0.05$; *, $p < 0.05$; **, $p < 0.01$; ***, $p < 0.001$ as determined by ANOVA).

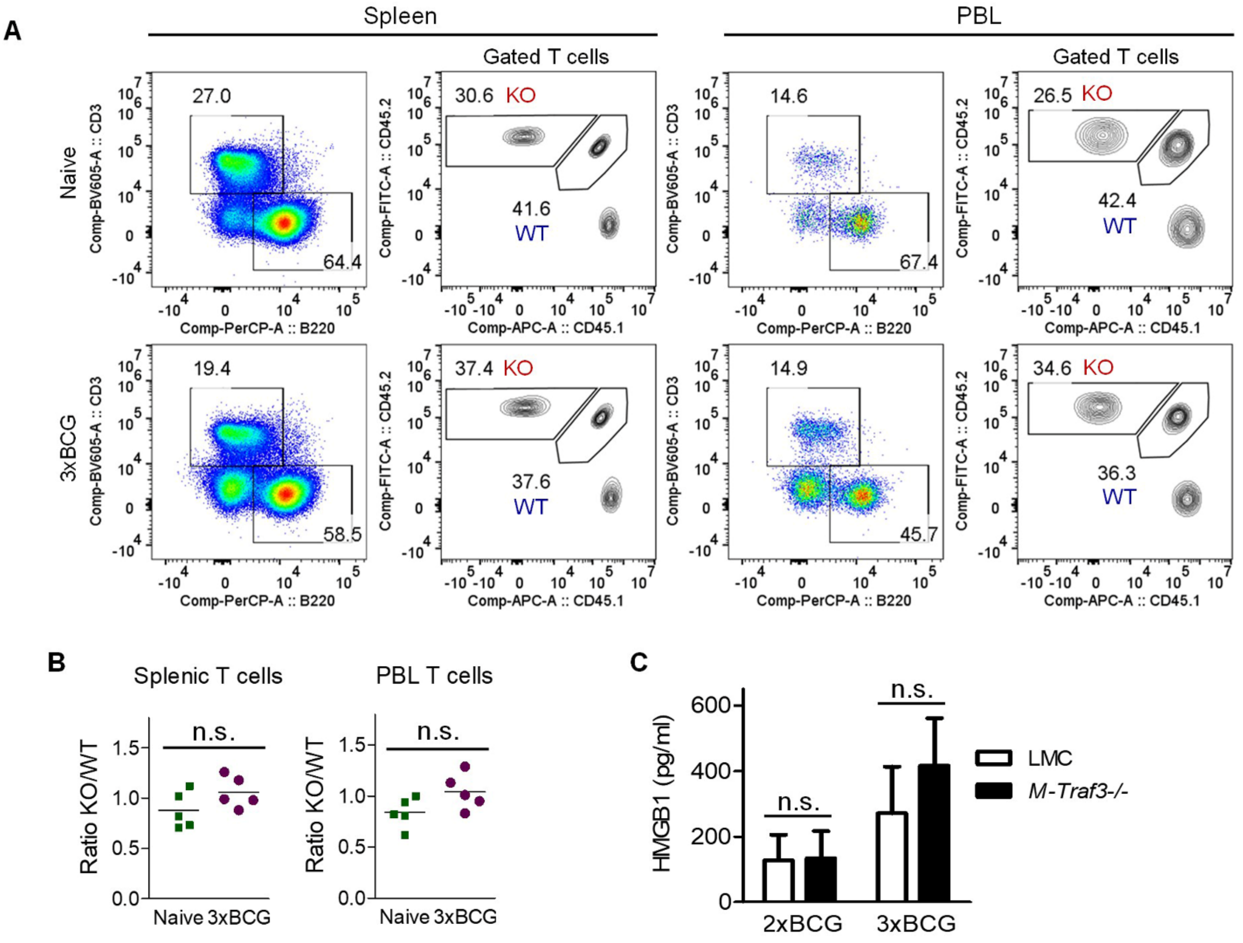

Figure S5. FACS analyses of T cells in mixed BM chimeras with or without repeated injections of heat-killed BCG. BM cells derived from gender-matched, young adult WT (CD45.1/CD45.2) and M-*Traf3*$^{-/-}$ (CD45.2) mice were mixed at a 1:1 ratio and adoptively transferred into lethally irradiated C57.SJL (CD45.1) recipient mice. At 8 weeks after reconstitution, a cohort of mixed BM chimeras were subjected to repeated injections of heat-killed BCG. On day 2 after the 3rd BCG injection (3xBCG), the BCG-treated and naïve chimeras that were generated from the same experiments were harvested and analyzed together. (**A**) Representative FACS profiles of M-*Traf3*$^{-/-}$ (KO, CD45.2+CD45.1-)-derived and WT (CD45.1+CD45.2+)-derived T cells (CD3+B220-CD11b- gated) of the spleen and PBL in naïve *versus* BCG-treated mixed BM chimeras.
(**B**) Graphical results comparing the ratio of KO/WT of gated T cells (CD3+B220-CD11b-) in the spleen and PBL of naïve *versus* BCG-treated mixed BM chimeras (n=5/group; n.s., $p > 0.05$ as determined by *t* test). (**C**) Serum levels of HMGB1 measured by ELISA (n=6/group; n.s., $p > 0.05$ as determined by ANOVA). Sera were collected from gender-matched, young adult LMC and M-*Traf3*$^{-/-}$ mice at day 2 after the 2nd (2xBCG) or 3rd (3xBCG) injection with heat-killed BCG.

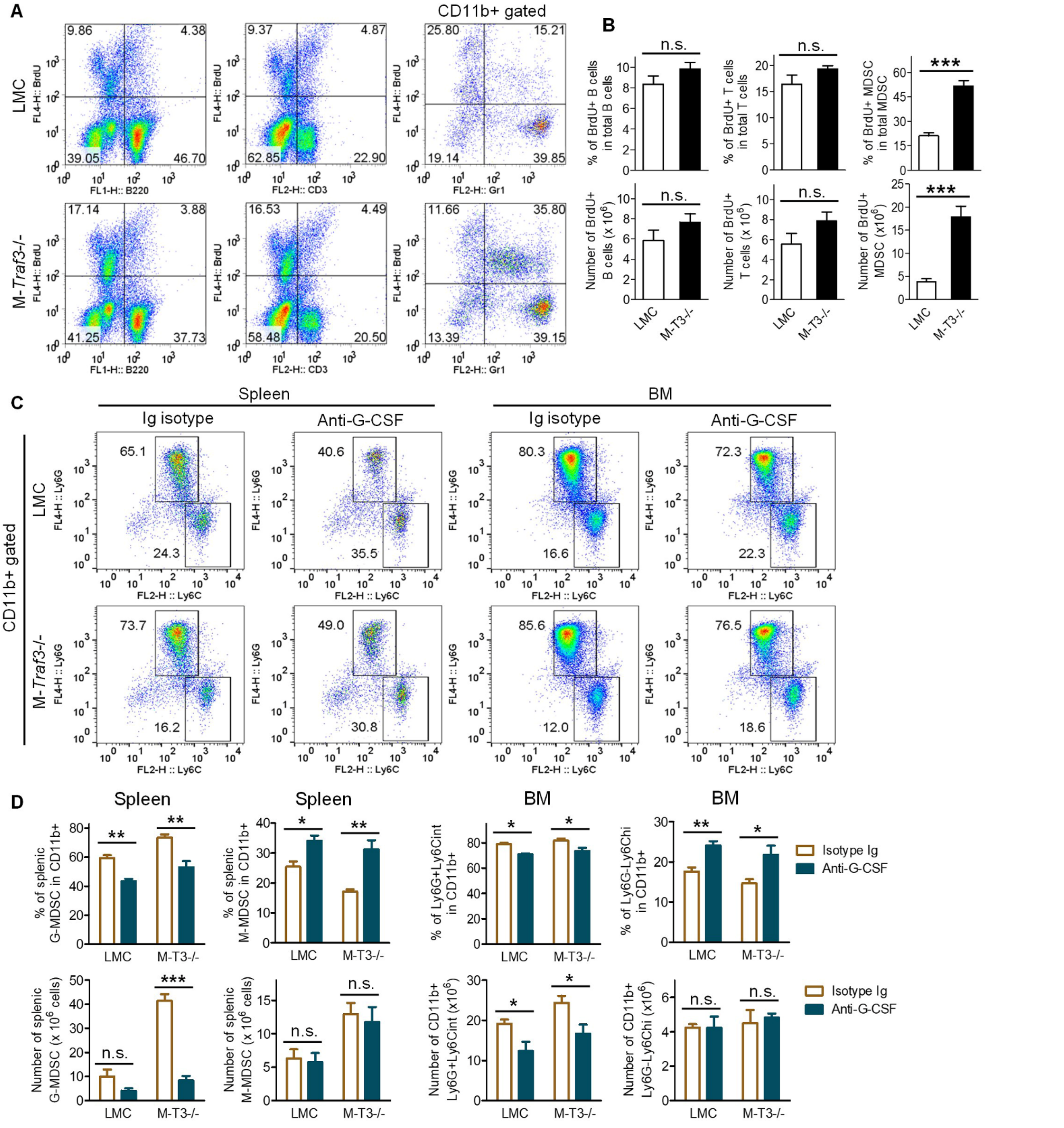

Figure S6. Increased frequency of proliferating MDSCs in the spleen and inhibition of G-MDSC expansion by *in vivo* neutralization of G-CSF in BCG-treated M-*Traf3*$^{-/-}$ mice. Gender-matched, young adult LMC and M-*Traf3*$^{-/-}$ mice (2-3-month-old) were subjected to repeated *s.c.* injections of heat-killed BCG at a 1-week interval. (**A** and **B**) At day 1 after the 2nd BCG injection, mice were injected *i.p.* with 3 mg BrdU in PBS twice at 19 h and 16 h before sacrifice. Mice were euthanized on day 2 after the 2nd BCG injection. (**A**) Representative FACS profiles of proliferating B cells (B220+BrdU+), T cells (CD3+BrdU+), and MDSCs (CD11b+Gr1+BrdU+) in the spleen of LMC and M-*Traf3*$^{-/-}$ mice. (**B**) Graphical results of the percentage and number of proliferating (BrdU+) B cells, T cells, and MDSCs in the spleen of LMC and M-*Traf3*$^{-/-}$ mice as determined by FACS (n=6/group). (**C** and **D**) Beginning at day 1 after the 1st BCG injection, mice were injected *i.p.* with 25 µg of anti-G-CSF neutralizing antibody or Ig isotype control thrice weekly for 2 weeks. Mice were dissected on day 2 after the 3rd BCG injection. (**C**) Representative FACS profiles of Ly6C and Ly6G staining on CD11b+ gated splenocytes and BM cells of LMC and M-*Traf3*$^{-/-}$ mice. Gated populations indicate CD11b+Ly6G+Ly6C^{int} G-MDSCs and CD11b+Ly6G-Ly6C^{hi} M-MDSCs. (**D**) Graphical results of the percentage and number of gated G-MDSCs (CD11b+Ly6G+Ly6C^{int}) and M-MDSCs (CD11b+Ly6G-Ly6C^{hi}) in the spleen and BM of LMC and M-*Traf3*$^{-/-}$ (M-T3-/-) mice obtained from 3 experiments. Graphs (B and D) depict the mean ± SEM (n.s., $p > 0.05$; *, $p < 0.05$; **, $p < 0.01$; ***, $p < 0.001$). The p values were determined by t test (B) or ANOVA (D).

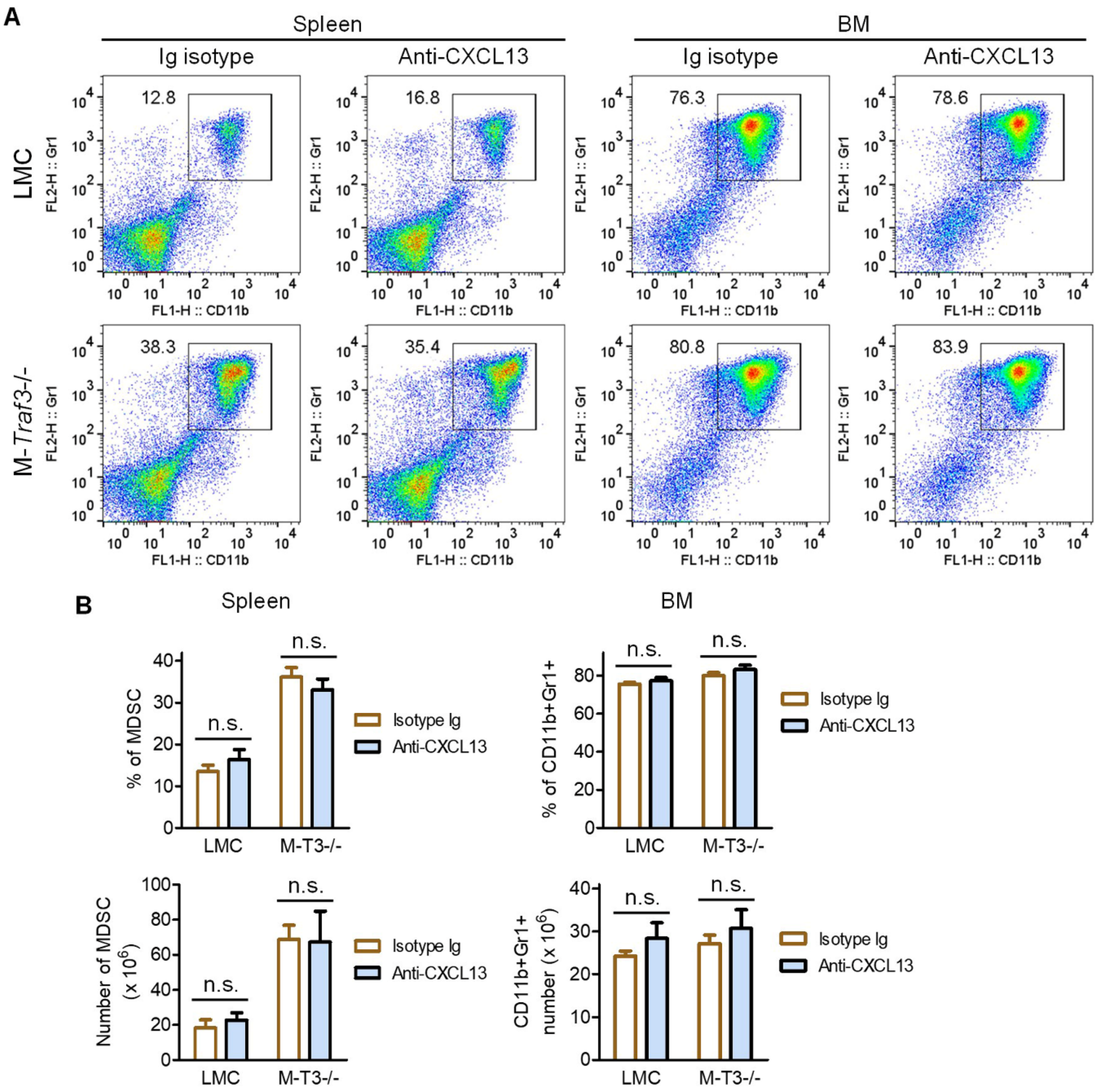

Figure S7. *In vivo* neutralization of CXCL-13 did not suppress the expansion of MDSCs in BCG-treated M-*Traf3*$^{-/-}$ mice. Gender-matched, young adult LMC and M-*Traf3*$^{-/-}$ mice (2-4-month-old) were subjected to repeated *s.c.* injections of heat-killed BCG. Beginning at day 1 after the 1st BCG injection, mice were injected *i.p.* with 10 µg of anti-CXCL-13 neutralizing antibody or Ig isotype control thrice weekly for 2 weeks. Mice were dissected on day 2 after the 3rd BCG injection. (**A**) Representative FACS profiles of CD11b and Gr1 staining on splenocytes and BM cells of LMC and M-*Traf3*$^{-/-}$ mice. (**B**) Graphical results of the percentage and number of CD11b+Gr1+ cells in the spleen and BM of LMC and M-*Traf3*$^{-/-}$ (M-T3-/-) mice obtained from 3 experiments (mean ± SEM; n.s., $p > 0.05$ as determined by ANOVA).

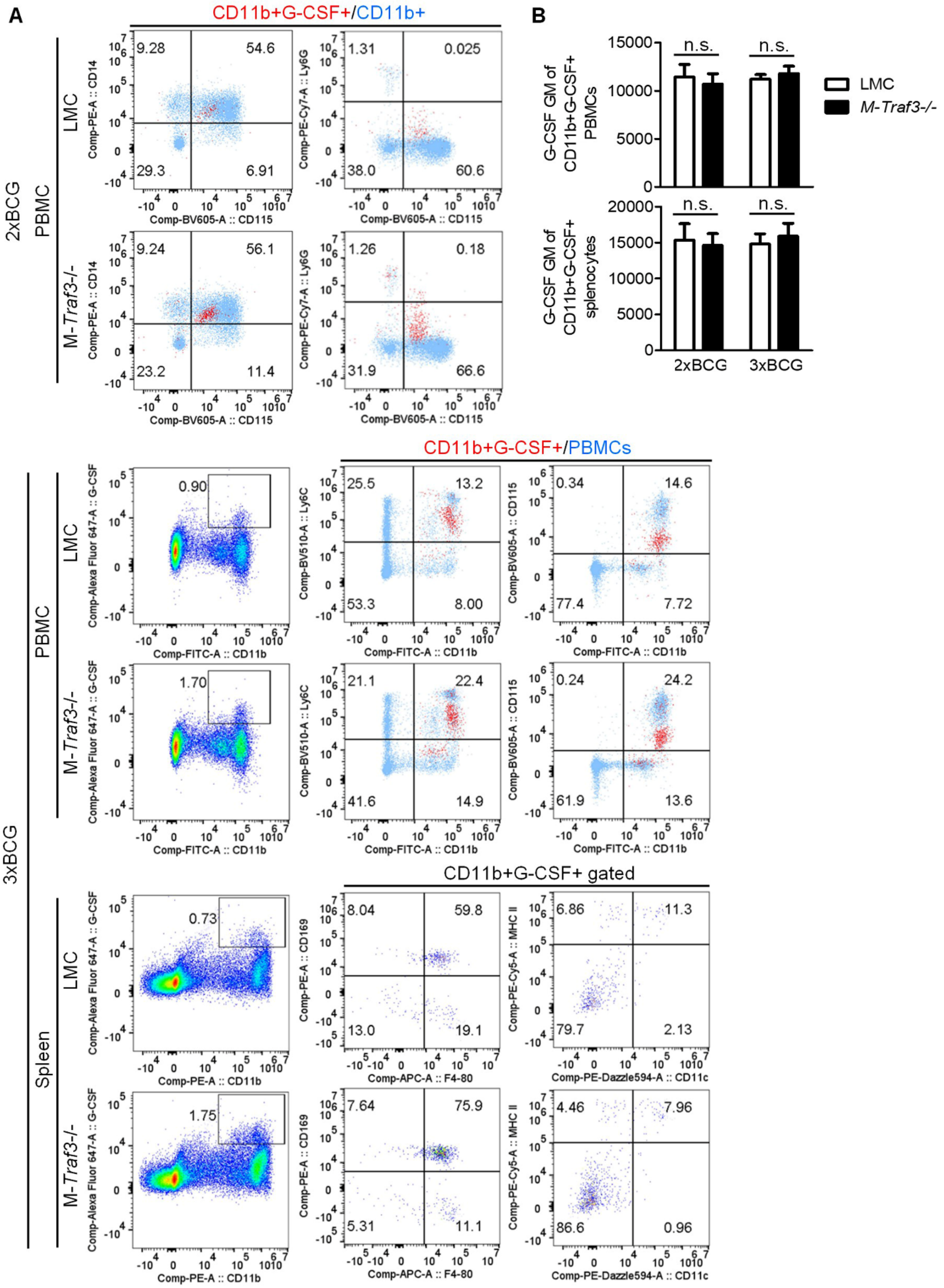

Figure S8. G-CSF-producing cells in BCG-treated LMC and M-*Traf3*$^{-/-}$ mice. PBMCs and splenocytes were harvested from gender-matched, young adult LMC and M-*Traf3*$^{-/-}$ mice at day 1 after the 2nd (2xBCG) or 3rd BCG (3xBCG) injection. (**A**) Representative FACS profiles of CD11b and G-CSF staining on PBMCs and splenocytes of LMC and M-*Traf3*$^{-/-}$ mice. For PBMCs harvested after the 2nd (2xBCG) injection, CD11b+G-CSF+ gated cells (red) were overlaid on top of CD11b+ gated PBMCs (light blue) to compare their expression of CD115, CD14, and Ly6G. For PBMCs harvested after the 3rd BCG (3xBCG) injection, CD11b+G-CSF+ gated cells (red) were overlaid on top of ungated PBMCs (light blue) to compare their expression of Ly6C and CD115. For splenocytes harvested after the 3rd BCG (3xBCG) injection, CD11b+G-CSF+ gated cells were further analyzed for their expression of F4/80 *versus* CD169 and CD11c *versus* MHC class II. (**B**) Graphical results of the geometric mean (GM) of G-CSF staining intensity on CD11b+G-CSF+ gated PBMCs and splenocytes (n=6/group) of LMC and M-*Traf3*$^{-/-}$ mice. Graphs depict the mean ± SEM (n.s., $p > 0.05$ as determined by ANOVA).

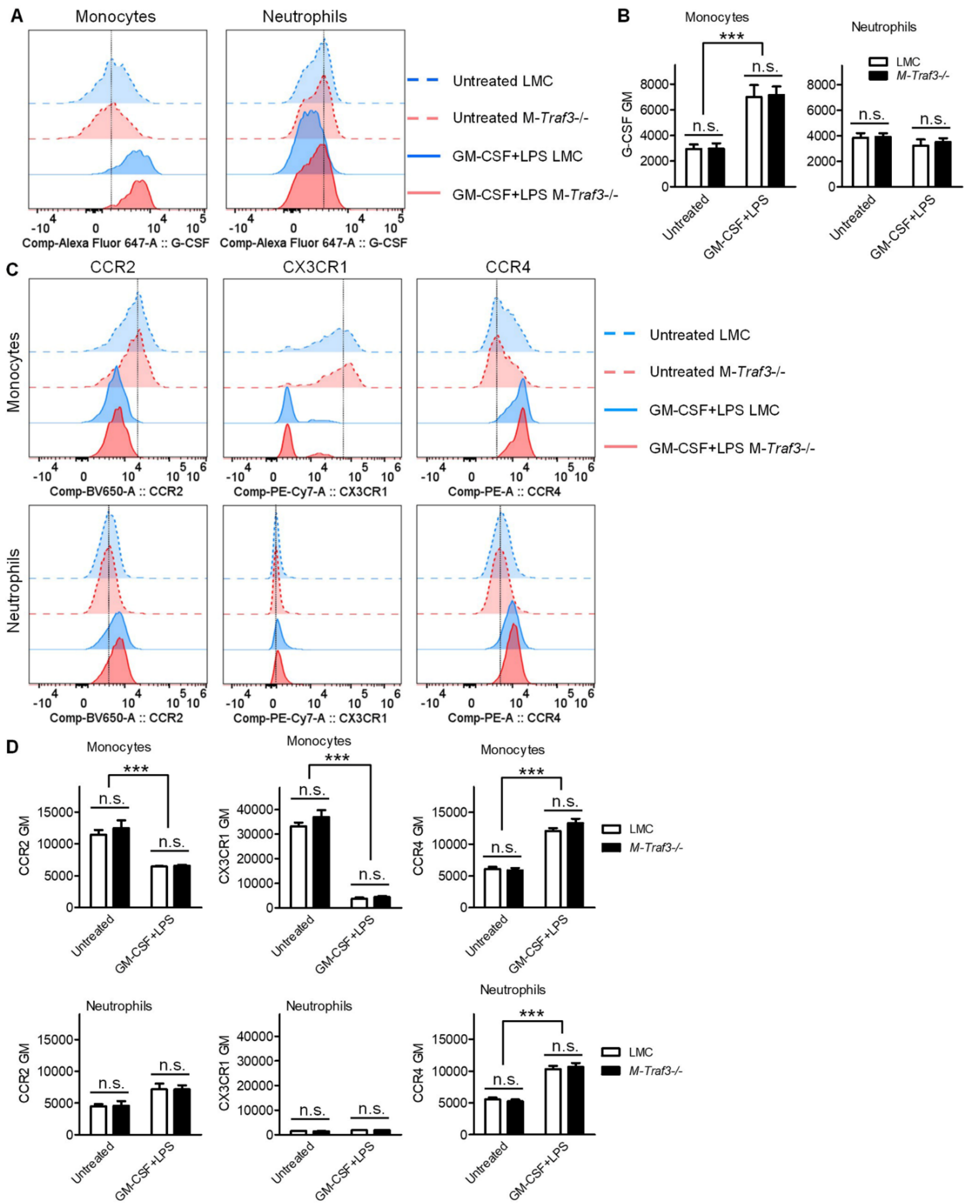

Figure S9. *In vitro* LPS-induced G-CSF production and chemokine receptor expression in cultured BM monocytes and neutrophils. BM cells were harvested from gender-matched, young adult naïve LMC and M-*Traf3*$^{-/-}$ mice. Cells were cultured in the absence or presence of 10 ng/ml GM-CSF and 100 ng/ml LPS for 24 h, and then production of G-CSF and expression of chemokine receptors in monocytes/monocytic lineage cells and neutrophils/granulocytic lineage cells were analyzed by FACS. Monocytes/monocytic lineage cells were gated as B220-CD11b+Ly6G-Ly6C+CD115+, while neutrophils/granulocytic lineage cells were gated as B220-CD11b+CD115-Ly6G+Ly6C+. (**A**) Example FACS histogram overlay comparing the levels of G-CSF staining intensity in gated monocytes and neutrophils after culture. (**B**) Graphical results of the geometric mean (GM) of G-CSF staining intensity in gated monocytes and neutrophils. (**C**) Representative FACS histogram overlay comparing the expression levels of CCR2, CX3CR1 and CCR4 on gated monocytes and neutrophils after culture. (**D**) Graphical results of the geometric mean (GM) of CCR2, CX3CR1 and CCR4 staining intensity on gated monocytes and neutrophils. Graphs (B and D) depict the mean ± SEM (n=4/group; n.s., $p > 0.05$; ***, $p < 0.001$ as determined by ANOVA).

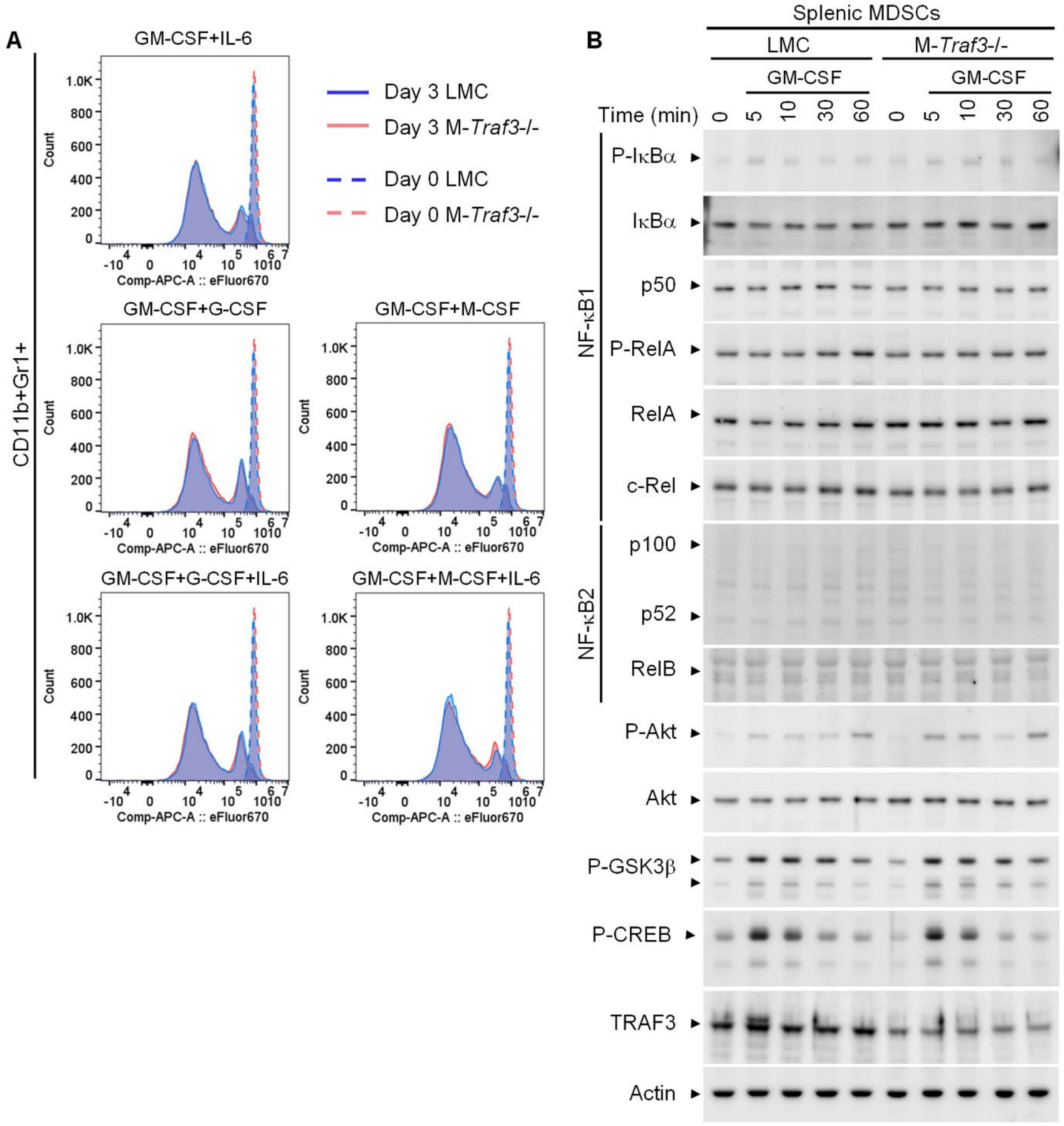

Figure S10. Proliferation of CD11b+Gr1+ BM cells and GM-CSF-induced early signaling events in splenic MDSCs. (**A**) BM cells were harvested from gender-matched, young adult naïve LMC and M-*Traf3*$^{-/-}$ mice, labeled with the cell proliferation dye eFluor 670 and cultured in the presence of 10 ng/ml GM-CSF in combination with 10 ng/ml of IL-6, G-CSF or M-CSF for 3 days. Representative FACS histogram overlay showing similar dilution of the labeled proliferation dye in CD11b+Gr1+ gated BM cells of the two genotypes after treatment with the indicated stimuli. (**B**) Splenic MDSCs were purified from gender-matched, young adult LMC and M-*Traf3*$^{-/-}$ mice at day 2 after the 3rd injection with heat-killed BCG. Purified MDSCs were serum-starved for 2 h, and then stimulated with 10 ng/ml GM-CSF for the indicated time (5-60 min). Total protein lysates were immunoblotted for phosphorylated (P-) or total IκBα, p50 NF-κB1, RelA, c-Rel, p100/p52 NF-κB2, RelB, Akt, GSK3β, and CREB followed by TRAF3 and actin. Results shown in (A) and (B) are representative of 3 experiments.
